# Investigating Apoptozole as a Chemical Probe for HSP70 Inhibition

**DOI:** 10.1371/journal.pone.0140006

**Published:** 2015-10-12

**Authors:** Lindsay E. Evans, Matthew D. Cheeseman, Norhakim Yahya, Keith Jones

**Affiliations:** Cancer Research UK Cancer Therapeutics Unit at The Institute of Cancer Research, London SW7 3RP, United Kingdom; Boston University Medical School, UNITED STATES

## Abstract

The use of chemical tools to validate clinical targets has gained in popularity over recent years and the importance of understanding the activity, selectivity and mechanism of action of these compounds is well recognized. Dysregulation of the HSP70 protein family has been linked to multiple cancer types and drug resistance, highlighting their importance as popular targets for anti-cancer drug development. Apoptozole is a recently identified small molecule, which has been reported to possess strong affinity for the HSP70 isoforms HSP72 and HSC70. We investigated apoptozole as a potential chemical tool for HSP70 inhibition. Unfortunately, using both biochemical and biophysical techniques, we were unable to find any experimental evidence that apoptozole binds to HSP70 in a specific and developable way. Instead, we provide experimental evidence that apoptozole forms aggregates under aqueous conditions that could interact with HSP70 proteins in a non-specific manner.

## Introduction

In recent years, lack of efficacy has been one of the most common causes of drug candidates failing clinical trials.[1–4] The 2014 report by Astra Zeneca, analyzing their small molecule drug discovery programs over a five year period, found that the primary cause of drug candidates failing clinical trails due to lack of efficacy to be when “pharmacological engagement of a proposed mechanism of action did not result in clinical benefit in the patient population tested”.[2] In response to this problem the use of chemical tools to validate clinical targets has gained in popularity. Chemical tools are small molecules that are used to understand the function of genes and proteins and to probe their role in physiology and pathology.[5–8] Chemical tools can be used to validate the connection between modulating the activity of a target protein and the observed biological effect, and confirm that modulation of protein function by small molecules can phenocopy genetic experiments, such as siRNA knockdown. The sequencing of the human genome and the subsequent increased interest in targeted drug therapies has made the use of chemical probes especially pertinent in disease areas such as oncology.[5] However, it is extremely important that the activity, selectivity and mechanism of action is well understood for tool compounds, so that the correct interpretations of biology and pharmacology can be made using these molecules.[5,6,9] This requirement is much more stringent than for potential drug compounds, where a balance of properties is required to progress a compound into the clinic and polypharmacology may be an acceptable contributor to efficacy.[10]

The 70 kDa heat shock protein family (HSP70s) are molecular chaperones responsible for maintaining protein homeostasis by protecting cells against stress-induced protein misfolding and aggregation.[11–13] The dysregulation of HSP70 chaperones has been implicated in a number of different disease areas including neurodegeneration, infection and cancer.[14,15] In cancer cells, increased levels of HSP70 proteins are thought to aid survival in the high-stress environment of the tumour.[16,17] The upregulation of HSP70 has also been identified as a possible resistance mechanism to HSP90 inhibition by small molecules; a number of which are currently undergoing clinical trials for the treatment of cancer.[16] RNAi knockdown of HSP70 has been shown to increase the efficacy of HSP90 inhibitors *in vitro* and dual HSP70/HSP90 treatment has been proposed as a potential solution to the resistance problem.[18,19] Recent studies have shown that the depletion of both the constitutively active HSC70 (HSPA8) and the stress-inducible HSP72 (HSPA1A, HSPA1B) isoforms is required to induce apoptosis in cancer cell lines, without causing effects in non-malignant cells.[19,20] It has therefore been predicted that inhibition of both protein isoforms would be required to produce a therapeutic effect in humans but this is yet to be confirmed using small molecules.[11]

**Fig 1:**
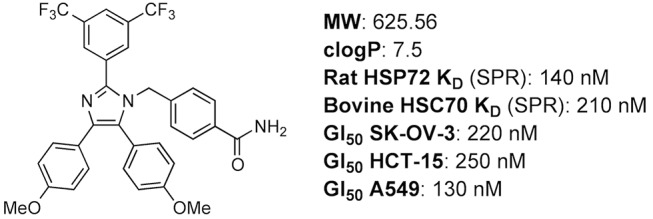
Chemical structure[26] and summary of physical properties and reported biochemical and cellular activity of apoptozole [25].

Several different approaches have been taken in attempts to identify small molecule modulators of HSP70, including targeting the ATP-binding site using structure-based design,[21] high-throughput screening to identify modulators of HSP70 ATPase activity[22] and targeting of the substrate binding domain (SBD) using peptidomimetics.[23] HSP70 remains a challenging target for drug development, due in part to its complex catalytic cycle, flexible protein structure and high affinity for its endogenous ligand ADP.[24] In 2008, the discovery of a small molecule with activity against both HSC70 and HSP72 was reported.[25] The biologically-active, tetrasubstituted imidazole, which was subsequently named apoptozole (Fig 1),[26] was identified during a cell-based screen designed to find compounds that induced apoptosis. The molecular target of apoptozole was identified as HSC70 in pull down experiments, though the site of interaction remains unknown. The binding affinity of apoptozole for rat HSP72 and bovine HSC70 was then determined by SPR and reported as 140 and 210 nM, respectively.[25,27–29]

The reported nanomolar binding affinity for both HSP70 and HSC70 makes apoptozole an attractive start point for inhibitor development. The aims of this work were to use apoptozole as a tool compound to investigate the physiological effect of dual HSP72/HSC70 inhibition with a small molecule and to establish an assay capable of screening for inhibitors with the same mode of action but improved physicochemical properties. Initially, we attempted to examine the binding affinity of apoptozole for human isoforms of HSP70 and investigate the site of interaction between apoptozole and HSP70 using a fluorescence polarisation (FP) assay. Interaction between apoptozole and human HSP70 was also analyzed by surface plasmon resonance (SPR) and the aggregation behaviour of apoptozole under aqueous conditions examined by dynamic light scattering (DLS).

## Materials and Methods

### Proteins and Inhibitors

The human HSPA1A (HSP72) gene was amplified from the IMAGE clone 3345864 (accession# BC002453) by PCR using the respective forward and reverse primers 5’-GATCGACCATATGGCCAAAGCCGCGGCGA-3’ and 5’-ACAGAATTCCTAATCTACCTCCTCAATGG-3’. The HSPA1A gene was cloned into a pTWOE vector, which is a modified version of pET-17b (Merck Chemicals Ltd, Nottingham, UK) encoding a *N*-terminal 6 x His-tag followed by a human rhinovirus 3C protease cleavage site. BL21-AI™ cells (Invitrogen, Paisley, UK) transformed with the vector containing the HSPA1A gene were grown in Luria-Bertani medium to an optical density at 600 nm of 0.6 and induced with 0.5 mM isopropyl-β-D-1-thiogalactopyranoside and 0.2% (*w/v*) arabinose at for 16 hours at 20°C. Cells were harvested by centrifugation at 6,000 RPM for 40 minutes at 4°C using an Avanti centrifuge J-26XP (Beckman Coulter™, High Wycombe, UK) with a JLA 8.100 rotor. Cell pellets were resuspended in 3 volumes of Lysis buffer consisting of 25 mM Tris, 50 mM NaCl, 5% (*v/v*) glycerol, 1 x cOmplete™ EDTA-free protease inhibitors (Roche, Basel, Switzerland), 25 U/mL Benzonase ® nuclease (Merck Chemicals Ltd) at pH 7.5. Cell lysis was performed by sonication using a Vibra-Cell^TM^ VCX500 (Sonics & Materials Inc, Newtown, USA) with a 13 mm solid probe for 24 cycles of 5 second on, 55 second off with amplitude set at 50%. The lysate was clarified by centrifugation at 20,000 RPM for 30 minutes at 4°C using an Avanti centrifuge J-26XP (Beckman Coulter™) with a JA 25.50 rotor.

The supernatant was passed through a 1.2 μm syringe filter (Sartorius Stedim, Germany) and loaded onto a 5 mL Histrap FF column (GE Healthcare, Chalfront St. Giles, UK) equilibrated in Buffer A, comprising 25 mM Tris, 50 mM NaCl, pH 7.5, and eluted with a gradient of 0–100% Buffer B (Buffer A + 250 mM Imidazole) over 10 column volumes (CV). Fractions containing HSP72 were pooled, concentrated, and loaded onto a Superdex 200 (16/60) size exclusion column (GE Healthcare) equilibrated in 25 mM Tris, 400 mM NaCl, 2 mM EDTA 5% (*v/v*) glycerol, pH 7.5 for further purification. Fractions containing HSP72 were pooled and further purified to remove contaminating nucleotides using a 6 mL Resource^TM^ Q column (GE Healthcare) equilibrated in 20 mM Tris, 2 mM EDTA, 5% (*v/v*) glycerol, pH 7.5. Following a 10 CV wash with the same buffer, HSP72 was eluted using a gradient from 0 to 500 mM NaCl over 6 CV and loaded onto a Superdex 200 16/60 column (GE Healthcare) equilibrated in a buffer containing 25 mM Tris, 400 mM NaCl, 15 mM EDTA, 5% (*v/v*) glycerol, pH 7.5. The removal of contaminating nucleotides was followed by measuring the ratio of absorbance at 260 nm and 280 nm (A_260_/A_280_) using a NanoDrop ND-1000 UV spectrophotometer (Thermofisher,Wilmington, USA). Samples with A_260_/A_280_ below 0.6 were regarded as nucleotide free.

Rat HSP72 protein was purchased from Enzo Life Sciences, human HSC70 was purchased from Abcam. EDC, NHS, ethanolamine and neutravidin were purchased from GE Healthcare. VER-155008, ATP, ADP and 200 nm polystyrene beads were purchased from Sigma. N^6^-(6-Amino)hexyl-ATP-6-FAM (ATP-FAM) and N^6^-(6-Amino)hexyl-ATP-Cy5 (ATP-Cy5) (1.0 mM in aqueous) were purchased from Jena Bioscience. Proteins and chemicals purchased from commercial supplies were used as supplied without further purification. Details of chemical synthesis are given in S1 Appendix.

### Fluorescence polarization assay

Unless otherwise stated the aqueous assay buffer contained 50 mM TRIS base pH 7.4, 150 mM NaCl, 6 mM MgCl_2_, 1 mM DTT, 0.1% (wt/wt) CHAPS. The assay was conducted using 384 Plus F ProxiPlates (PerkinElmer) with a final assay volume of 10 μL. Plates were centrifuged at 1000 rpm for 1 minute prior to incubation and read using an 2103 Envision Multilable Plate Reader. Excitation and emission wavelengths used for green probes were 480 nm and 535 nm, respectively. Excitation and emission wavelengths used for red probes were 620 nm and 688 nm, respectively. Fluorescence polarisation was measured in units of millipolarization (mP). All experiments were performed in triplicate, except for experiments with ATP-Cy5 and apoptozole-Cy5, which were performed in duplicate. Data were plotted and analyzed using GraphPad Prism 6, graphical data represents the geometric mean ± standard error of the mean for a single representative experiment.

### K_D_ determination

To each well, 5 μL of probe molecule (20 nM in assay buffer) and increasing concentrations of HSP70 protein (5 μL, two-fold dilution series) were added. Fluorescence polarisation values for tracer control wells (10 nM probe in assay buffer only) were subtracted from each data point prior to data analysis. K_D_ determination was performed using non-linear regression analysis (GraphPad Prism 6, one site–specific binding model).

### Competitive binding experiments

Compounds (0.2 μL at 50 x screening concentration in DMSO) were dispensed using an ECHO 550 Liquid Handler (labcyte Inc.). To the corresponding wells was added, 5 μL of probe molecule (20 nM in assay buffer) and 5 μL of protein (two times their final concentration in assay buffer) to give a 50% bound fraction.[30] Tracer controls (10 nM probe molecule only) and bound tracer controls (10 nM probe in the presence of appropriate protein concentration) were included on each assay plate. IC_50_ determination was performed using non-linear least squares curve fitting (GraphPad Prism 6, log(inhibitor) vs. response—variable slope (four parameters)).

### Surface Plasmon Resonance

SPR analysis was performed on a Biacore T200 (GE Healthcare) at 25°C at a flow rate of 30 μL/min. The running buffer contained 1 x PBS and was prepared from 10 x concentrated PBS (GE Healthcare) on the day of use. Series S Sensor Chip CM5 was purchased from GE Healthcare, stored at -4°C and brought to room temperature prior to use.

### Procedure A

The sensor chip was activated by injection of a 1:1 mixture of 500 mM NHS and 200 mM EDC for 420 seconds. Neutravidin (50 μg/mL in 10 mM sodium acetate buffer, pH 5.5) was injected across the chip surface for 60 seconds, followed by injection of 1 mM ethanolamine (pH 8.5) for 420 seconds. Biotinylated-apoptozole **4** (100 μM in 1:1 DMSO/PBS) was injected over the chip surface for 420 seconds. Human and rat HSP72 (500 nM in PBS) were injected at concentrations of 31–500 nM, injection time: 60 seconds, dissociation time: 60 seconds. The control lane was subjected to the conditions described above but derivatization with biotinylated apoptozole **4** was omitted.

### Procedure B

The sensor chip was activated by injection of a 1:1 mixture of 500 mM NHS and 200 mM EDC for 420 seconds. Apoptozole derivative **6** (100 μM in 1:1 DMSO/PBS) was injected over the chip surface, to give immobilization levels of approximately 4000, 8000 and 12000 RU. Ethanolamine (1 mM, pH 8.5) was then injected over the chip surface for 420 seconds. Human HSP72 (32 μM in PBS) was injected at concentrations of 62 nM–32 μM, injection time: 60 seconds, dissociation time: 60 seconds. The control lane was subjected to the conditions described above but derivatization with apoptozole derivative **6** was omitted.

### Dynamic light scattering

Apoptozole and VER-155008 (10 mM in DMSO) were diluted in 1 x PBS buffer with a final DMSO concentration of 2%, ± 0.01% v/v Triton X-100. Measurements were performed using a Protein Solutions DynaPro (Wyatt Technology) at room temperature. Data were acquired and processed by the software Dynamics, version 6.10 and calculation of particle radius performed by the cumulant analysis tool. The detector angle was 90°, the laser power was set to 30% and the integration time was 150 s. Each radius value presents three independent measurements, values are quotes as arithmetic mean ± standard error of the mean.

## Results

### Apoptozole analogues are not competitive with ATP for binding to HSP70

**Fig 2:**
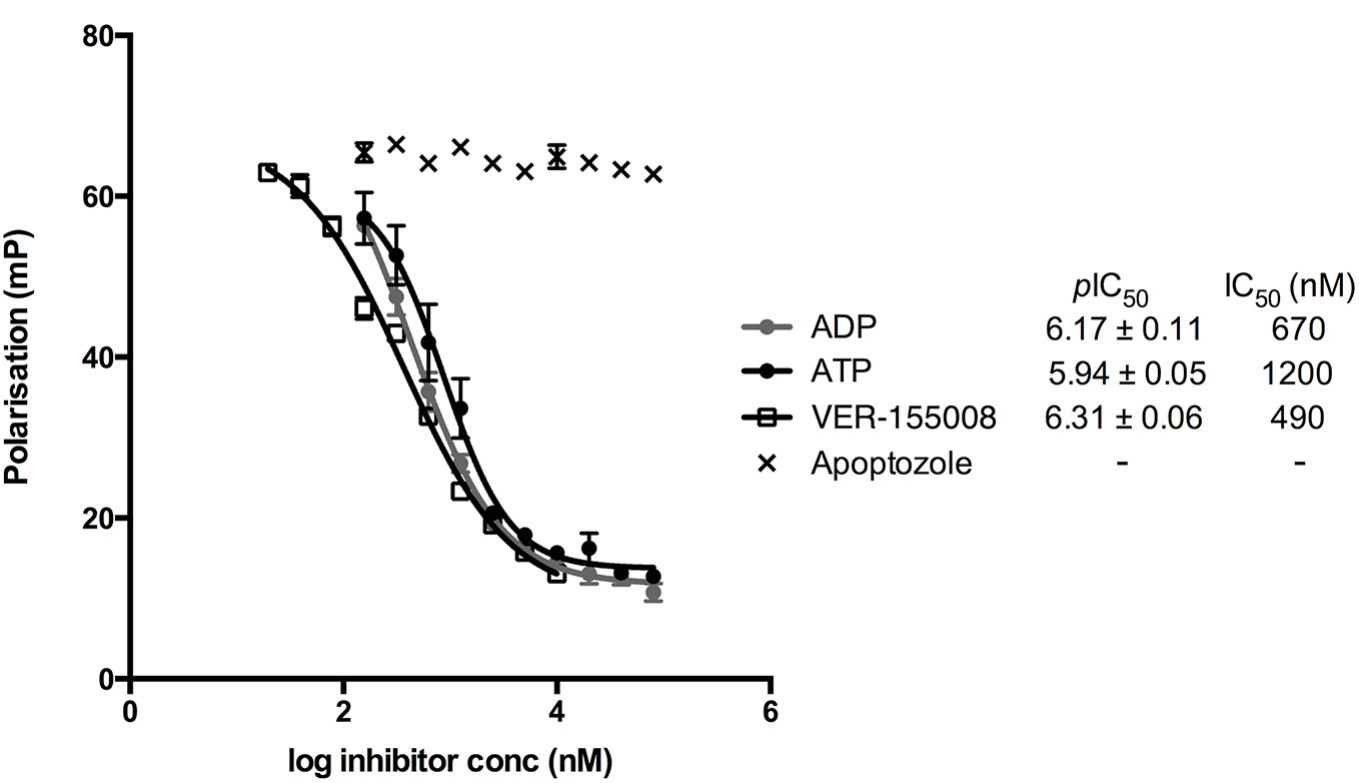
Competitive displacement curves for ATP-FAM and HSP72 with ATP, ADP, VER-155008 and apoptozole. IC_50_ values are the geometric mean and *p*IC_50_ values are the geometric mean ± SEM from 3 independent measurements.

We first addressed the binding site of apoptozole. Using reported evidence from ligand-directed protein labelling and molecular modelling, it has been proposed that apoptozole binds in the ATP-binding site of HSP70.[27] This would likely require apoptozole to be competitive with ATP for binding and for the binding of the ligands to be mutually exclusive. Using an ATP-derived fluorescent probe (ATP-FAM)[21] a fluorescence polarisation (FP) assay was developed to enable the detection of ATP-competitive inhibitors of human HSP72 (S1 and S2 Figs). IC_50_ determination was performed at the same time for the known ATP-competitive HSP70 inhibitor VER-155008 (S3 Fig)[21], and the endogenous ligands ATP and ADP. IC_50_ values of 490 nM, 1200 nM and 670 nM were determined for VER-155008, ATP and ADP, respectively. In contrast, apoptozole failed to show any measureable ATP-competitive binding to HSP72, with no displacement of the ATP-FAM probe observed at concentrations up to 80 μM (Fig 2). The lack of ATP-competitive binding strongly suggested that apoptozole was not binding in the ATP-binding site of HSP72.

### Fluorescent probes derived from apoptozole do not interact with HSP70 proteins

**Fig 3:**
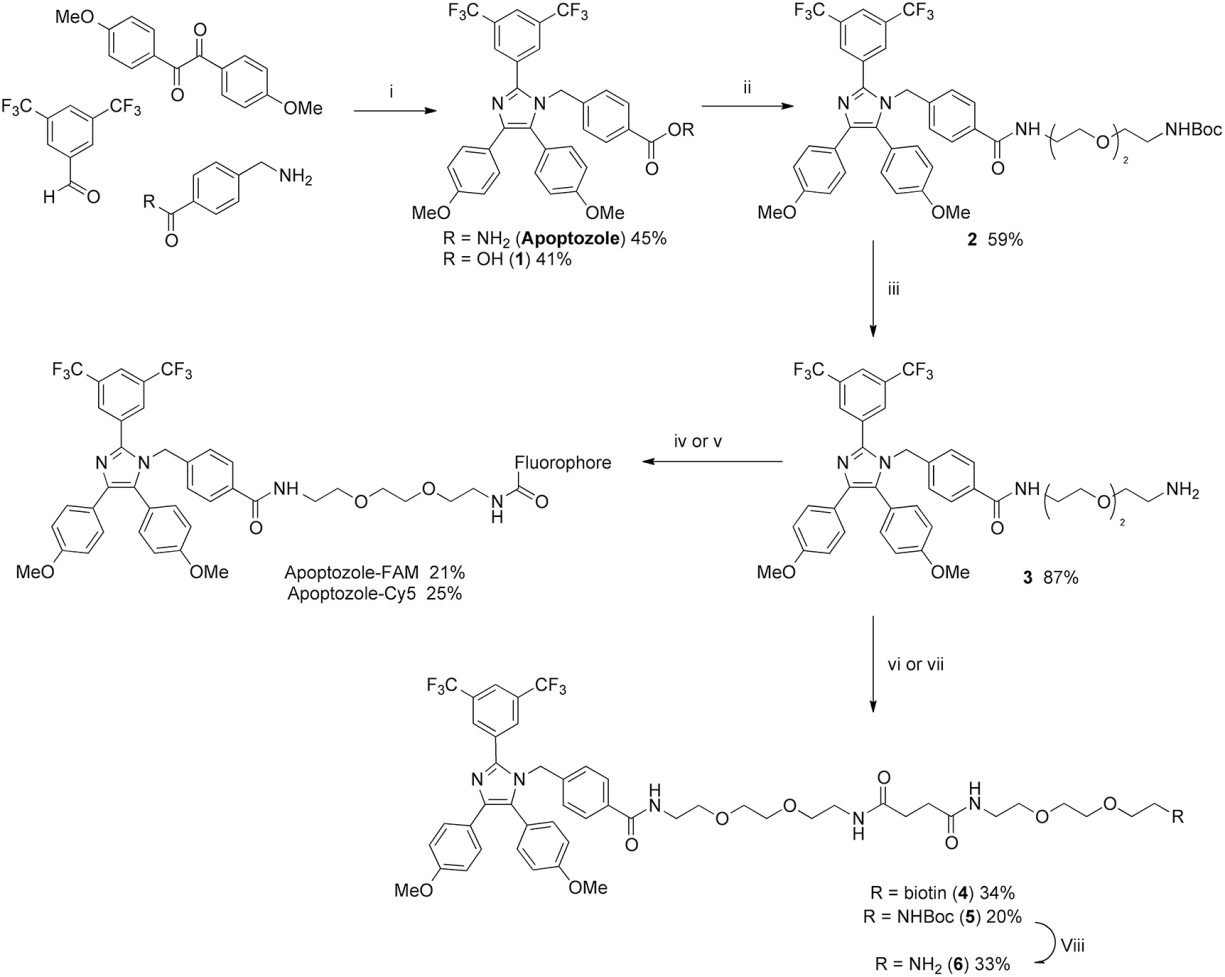
Chemical synthesis of apoptozole and derivatives. Reagents and conditions: i) NH_4_OAc, AcOH, 100°C, 16 hr; ii) *N*-Boc-3,6-dioxa-1,8-octanediamine, HBTU, DIPEA, DCM, rt, 5 hr; iii) 1:1 DMC/TFA, rt, 2 hr; iv) 5-FAM NHS ester, NEt_3_, DMF, rt, 16 hr; v) Cy5 NHS ester, NEt_3_, DMF, rt, 16 hr; vi) Biotin-derivative **7**, HBTU, DIPEA, DMF, rt, 1 hr; vii) Succinic anhydride, DIPEA, MeCN, 30°C, 16 hr, then *N*-boc-3,6-dioxa-1,8-octanediamine, HBTU, DIPEA, DMF, 35°C, 16 hr; viii) 1:1 DCM/TFA, rt, 16 hr.

Since apoptozole has been reported to interact with the nucleotide binding domain (NBD) of HSP70 proteins, the possibility remained that the site of interaction is remote from the nucleotide-binding pocket and that apoptozole is non-competitive with ATP. To further investigate the interactions between apoptozole and human HSP70 proteins, an FP assay using a probe derived from apoptozole was prepared. Two fluorescent probes derived from apoptozole, labelled with fluorescein (apoptozole-FAM) and Cy5 (apoptozole-Cy5), were designed and synthesised via the route outlined in Fig 3. For FP probes, the attachment site and length of the linker molecule used to connect the parent compound and fluorophore are critical. The fluorophore attachment site and choice of linker were informed by the work reported by Injae *et al*.[27] and the Cy5-labelled probe prepared to provide a close comparison with the Cy3-labelled apoptozole-derivative that Injae *et al*. used in cell-based studies.

**Fig 4:**
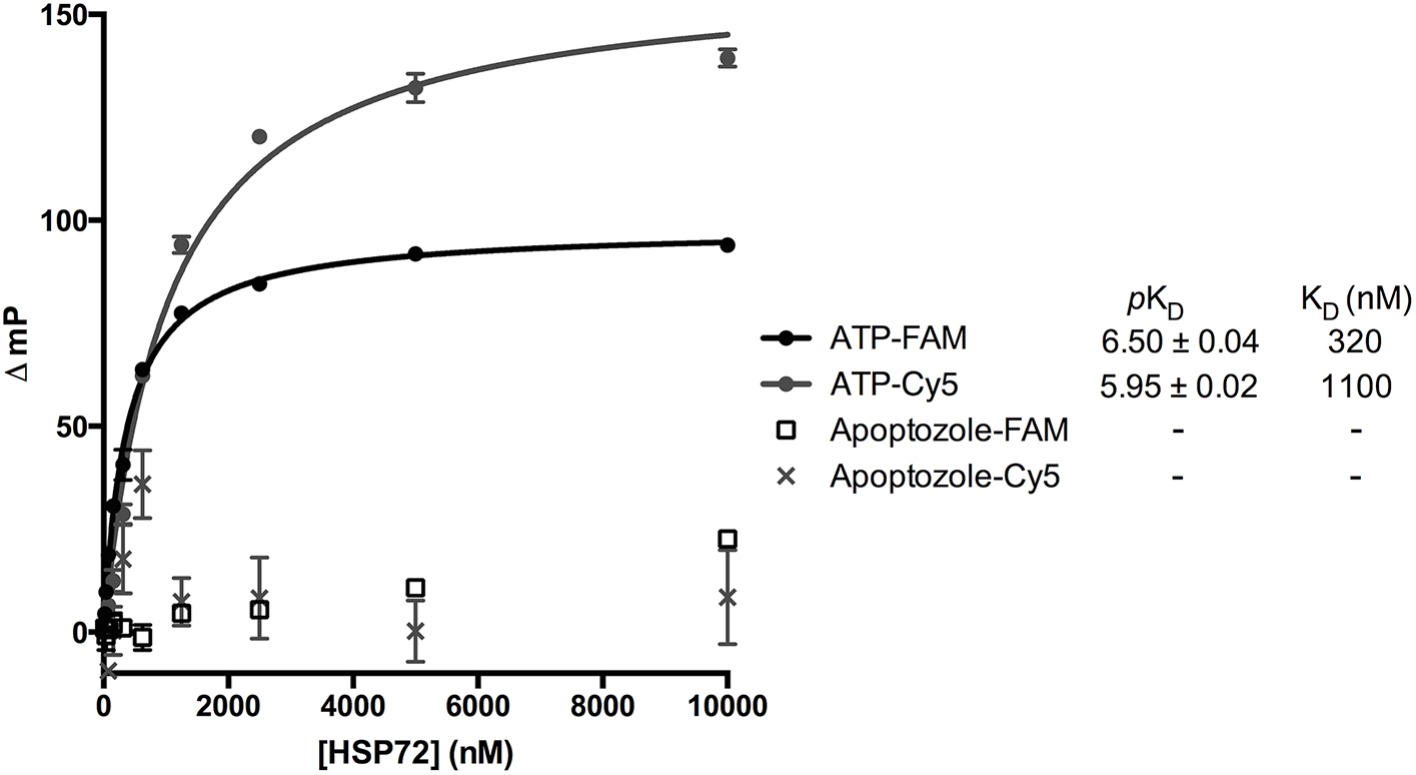
Binding isotherms for ATP-FAM, ATP-Cy5, apoptozole-FAM and apoptozole-Cy5 with HSP72. K_D_ values are the geometric mean and *p*K_D_ values are the geometric mean ± SEM from 3 independent measurements.

The binding affinities for HSP72 of apoptozole-FAM and apoptozole-Cy5 were determined by titrating increasing concentrations of human HSP72 against a fixed concentration (5 nM) of the probes. The experiment was performed using ATP-derivatives N^6^-(6-amino)hexyl-ATP-5-FAM (ATP-FAM) and N^6^-(6-amino)hexyl-ATP-Cy5 (ATP-Cy5) to provide positive controls (Fig 4). Non-linear regression analysis yielded K_D_ values of 320 nM and 1100 nM for ATP-FAM and ATP-Cy5, respectively. Unfortunately, no detectable binding was observed for either apoptozole-FAM or apoptozole-Cy5.

Fluorescence polarisation assays targeting both the NBD and SBD of HSP70 proteins are well precedented in the literature[21,31,32] and cell-based studies using fluorescently-labelled derivatives of apoptozole have also been reported.[27] It was therefore surprising that no interactions were observed, even at a protein concentration of 10 μM. Altering the assay conditions (metal ions, detergent and length of incubation) also failed to produce any detectable interactions between apoptozole-FAM and human HSP72 protein (S4 Fig). To examine if apoptozole could be isoform selective, K_D_ determinations for apoptozole-FAM and ATP-FAM were performed using human HSC70 (S5 Fig). Weak and non-saturating binding, indicative of non-specific interactions was detected for apoptozole-FAM, whilst a K_D_ value of 970 nM was determined for ATP-FAM.

### There is no evidence by SPR analysis that apoptozole interacts with HSP70

**Fig 5:**
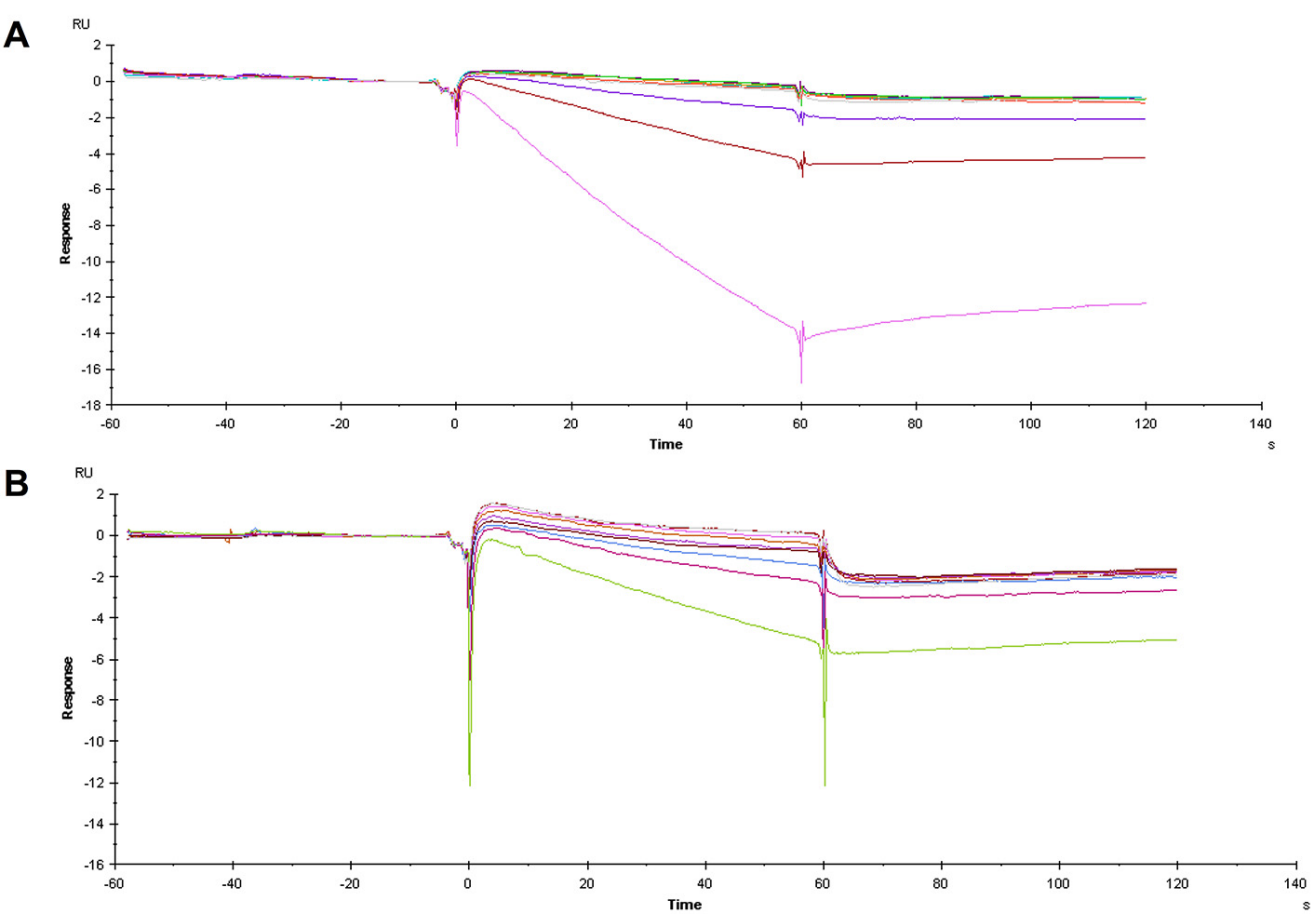
SPR sensorgrams of HSP72 interacting with biotinylated apoptozole 4 immobilised on a neutravidin derivatised CM5 sensor chip. (A) Human HSP72, control subtracted data; (B) Rat HSP72, control subtracted data.

Having failed to detect any specific binding interactions between either of the apoptozole-derived probes and human HSP70 proteins or to displace ATP-FAM with apoptozole, we were keen to explore other methods for investigating binding interactions between HSP72 and apoptozole. Injae *et al*. reported measuring the binding affinity of apoptozole for rat HSP72 and bovine HSC70 by SPR to give K_D_ values of 140 and 210 nM, respectively.[25] Despite the high sequence homology between the mammalian and human protein isoforms (human HSP72 is 96.6% identical to rat HSP72 and human HSC70 is 99.4% identical to bovine HSC70),[33] the possibility remained that apoptozole was not interacting with the human HSP70 proteins in the same manner. To investigate this, SPR experiments were performed using human and rat HSP72 protein according to the experimental procedure previously described.[25] The experiment was performed using an apoptozole-biotin conjugate **4** (Fig 3) immobilised onto a neutravidin-derivatized SPR chip. A lane containing only neutravidin functionalization was used as a binding control. Increasing concentrations of both human and rat HSP72 were flowed over the chip surface to produce the SPR traces shown in Fig 5. Unfortunately, there was no evidence of binding interactions occurring between either rat or human HSP72 and apoptozole, at any of protein concentrations tested.

**Fig 6:**
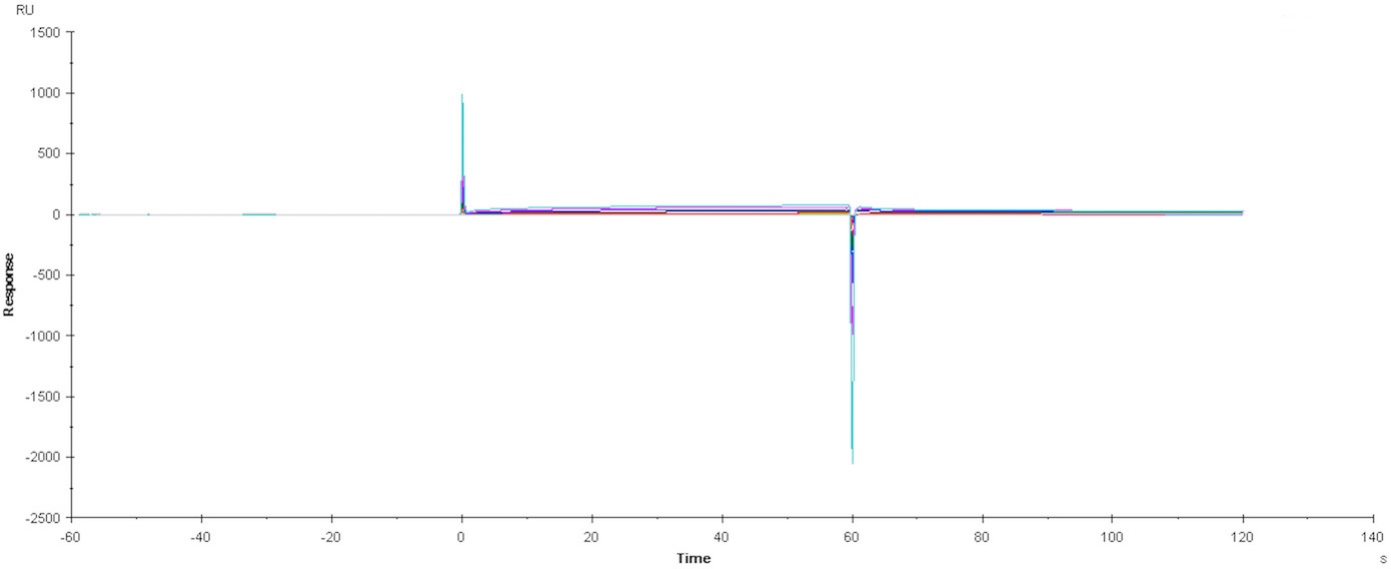
SPR sensorgram of human HSP72 interacting with CM5 sensor chip immobilised with apoptozole derivative 6.

Prior to subtraction of the neutravidin-derivatized control lane data from the binding data, the sensorgrams showed evidence of weak and non-saturating binding (S6 Fig) indicating non-specific binding interactions between HSP72 and the neutravidin-derivatised sensor chip. Subtraction of the control lane data from the experimental lane data produced the sensorgrams shown Fig 5A and 5B, which show no evidence of binding between apoptozole and rat or human HSP72. To investigate if interactions between HSP72 and neutravidin could be eclipsing the interactions between HSP72 and apoptozole, the experiment was repeated in the absence of neutravidin. An amine-derivative of apoptozole **6** (Fig 3) was designed to closely match the structure and linker length of biotinylated apoptozole **4**. Apoptozole derivative **6** was bound directly to the CM5 chip surface and the underivatised CM5 chip was used as the binding control. The experiment was performed at three different immobilisation levels, to remove any potential for interference from overloading of the chip surface. A representative example of the resulting sensorgrams is shown in Fig 6. For all three immobilisation levels no binding interactions were observed at any concentrations of HSP72 up to 32 μM. From this result, it was concluded that apoptozole was not interacting with recombinant rat or human HSP72 in a specific or measurable manner.

### Apoptozole aggregates under aqueous conditions

Using both biochemical and biophysical techniques, we had been unable to find evidence that apoptozole forms specific binding interactions with HSP70 proteins. However, results from biochemical, cellular and pull-down experiments using concentrations of apoptozole up to 500 μM have previously been reported.[25,27–29] Given the high molecular weight and high lipophilicity of apoptozole it seemed unlikely that this compound would behave favourably under aqueous conditions at such high concentrations. As a result, we hypothesised that the reported activity could be a result of assay interference rather than target engagement. It is widely known that small molecules, including many approved drugs, form aggregates in aqueous solutions and that these aggregates are capable of interacting with proteins and disrupting their function in both biochemical and cellular assays in a non-specific manner.[34–37] An imidazole ring combined with a high clogP value has previously been identified as a structural motif frequently found in molecules which are strong aggregate formers.[36] We investigated the behaviour of apoptozole under aqueous conditions using dynamic light scattering DLS, which enables the detection and quantification of molecular aggregation (Table 1). We found that at concentrations of apoptozole ≥ 5 μM, high light-scattering intensities and particles with a radius > 450 nm were detected. Addition of the non-ionic detergent Triton X-100 prevented the formation of the large particles. These observations are consistent with the formation of colloidal aggregates.[34] As a control, the experiment was also performed using VER-155008. VER-155008 showed no evidence of aggregate formation at 10 μM.

**Table 1:** Dynamic light scattering analysis of apoptozole and VER-155008.

	**Concentration (μM)**	**Triton X-100**	**Radius (nm)**	**Intensity (kcps)**
**PBS buffer**	N/A	-	4.5 ± 1.2	18.3 ± 0.6
	N/A	+	58.3 ± 3.5	221.5 ± 13.8
**100 nm bead**	N/A	-	60.5 ± 0.1	1694.3 ± 29.5
**Apoptozole**	10	-	521.5 ± 30.9	1441.1 ± 186.5
	10	+	86.5 ± 2.1	2350.0 ± 173.1
	5	-	454.4 ± 36.4	1047.5 ± 183.1
	1	-	89.3 ± 5.8	132.6 ± 39.7
**VER155008**	10	-	88.5 ± 8.3	63.2 ± 5.8
	10	+	101.1 ± 9.0	318.2 ± 61.3
	**Concentration (μM)**	**Triton X-100**	**Radius (nm)**	**Intensity (kcps)**
**PBS buffer**	N/A	-	4.5 ± 1.2	18.3 ± 0.6
	N/A	+	58.3 ± 3.5	221.5 ± 13.8
**100 nm bead**	N/A	-	60.5 ± 0.1	1694.3 ± 29.5
**Apoptozole**	10	-	521.5 ± 30.9	1441.1 ± 186.5
	10	+	86.5 ± 2.1	2350.0 ± 173.1
	5	-	454.4 ± 36.4	1047.5 ± 183.1
	1	-	89.3 ± 5.8	132.6 ± 39.7
**VER155008**	10	-	88.5 ± 8.3	63.2 ± 5.8
	10	+	101.1 ± 9.0	318.2 ± 61.3

Radius and intensity values are the arithmetic mean ± SEM from 3 independent experiments.

## Discussion

In this study we investigated the proposed small molecule inhibitor of HSP72 and HSC70 apoptozole. The requirement that both major cytosolic HSP70s be inhibited for a therapeutic effect to be observed has been reported by several groups, making apoptozole an interesting molecular target for further development. However, using both biochemical and biophysical techniques we were unable to find any experimental evidence that apoptozole interacts with HSP70 proteins in a specific and developable manner. Instead, apoptozole may form aggregates under aqueous conditions that could interact with HSP70 proteins in a non-specific manner, potentially leading to false positives and inconsistent data.

Results from cellular and pull-down experiments using concentrations of apoptozole up to 2 mM have been reported. In contrast, we found that under aqueous conditions apoptozole forms large colloidal aggregates at concentrations ≥ 5 μM. It is possible that some of the observed cellular effects of apoptozole *in vitro* are a result of aggregation. Our data shows that apoptozole is unlikely to be useful as a chemical tool for studying HSP70 inhibition or as a start point for inhibitor development. It has recently been reported that heat shock proteins, including HSP70, are frequently identified as false positives in pull-down experiments.[38] Another recent study, reported by Bukau, Mayer and co-workers, proposed that the previously reported inhibitor of HSP70 known as pifithrin-μ or PES, binds to HSP70 in an “*unspecific*, *detergent-like way*”.[20] PES was originally identified as an inhibitor of p53-mediated apoptosis[39] and like apoptozole, was identified as binding to HSP72 in a pull-down experiment.[40] These results suggest that HSP70 proteins are prone to non-specific binding of small molecules in both biochemical assays and pull-down experiments. As a result, the development of chemical tools to study HSP70 is extremely challenging and even more stringent criteria need to be applied to chemical tools to study this protein.

## Supporting Information

S1 Fig Determination of assay binding window for ATP-FAM. Polarization values (mP) for 2–31 nM ATP-FAM in the presence and absence of 5 μM HSP72. Assay was performed in triplicate and the mean and standard error plotted using GraphPad Prism 6.
(TIFF)

S2 Fig Binding isotherm for HSP72 and 10 nM ATP-FAM. Assay was performed in triplicate and the mean and standard error plotted using GraphPad Prism 6. K_D_ value is the geometric mean and *p*K_D_ values are the geometric mean ± SE from 3 independent measurements.
(TIFF)

S3 Fig Chemical structure of ATP-competitive HSP70 inhibitor VER-155008. (TIF)

S4 Fig Change in metal ions, detergent and length of incubation has no effect on binding of apoptozole-FAM to HSP72. Binding interaction not observed under any of the assay conditions tested, K_D_ values are for a single determination.
(TIFF)

S5 Fig Apoptozole-FAM does not bind to HSC70. K_D_ values are for a single determination.
(TIFF)

S6 Fig Surface Plasmon Resonance analysis of HSP72 binding to apoptozole prior to subtraction of control lane. Biotinylated apoptozole **4** was immobilized on a neutravidin-derivatised gold chip followed by injection of HSP72 (31–500 nM), individual protein concentrations are highlighted by colored traces.
(TIFF)

S1 Appendix Chemical Synthesis. (DOCX)
