# Supplementary figures and images for: Investigating Apoptozole as a Chemical Probe for HSP70 Inhibition
Source: PLoS One. 2015 Oct 12;10(10):e0140006. doi: 10.1371/journal.pone.0140006 (PMC4601772; doi:10.1371/journal.pone.0140006)

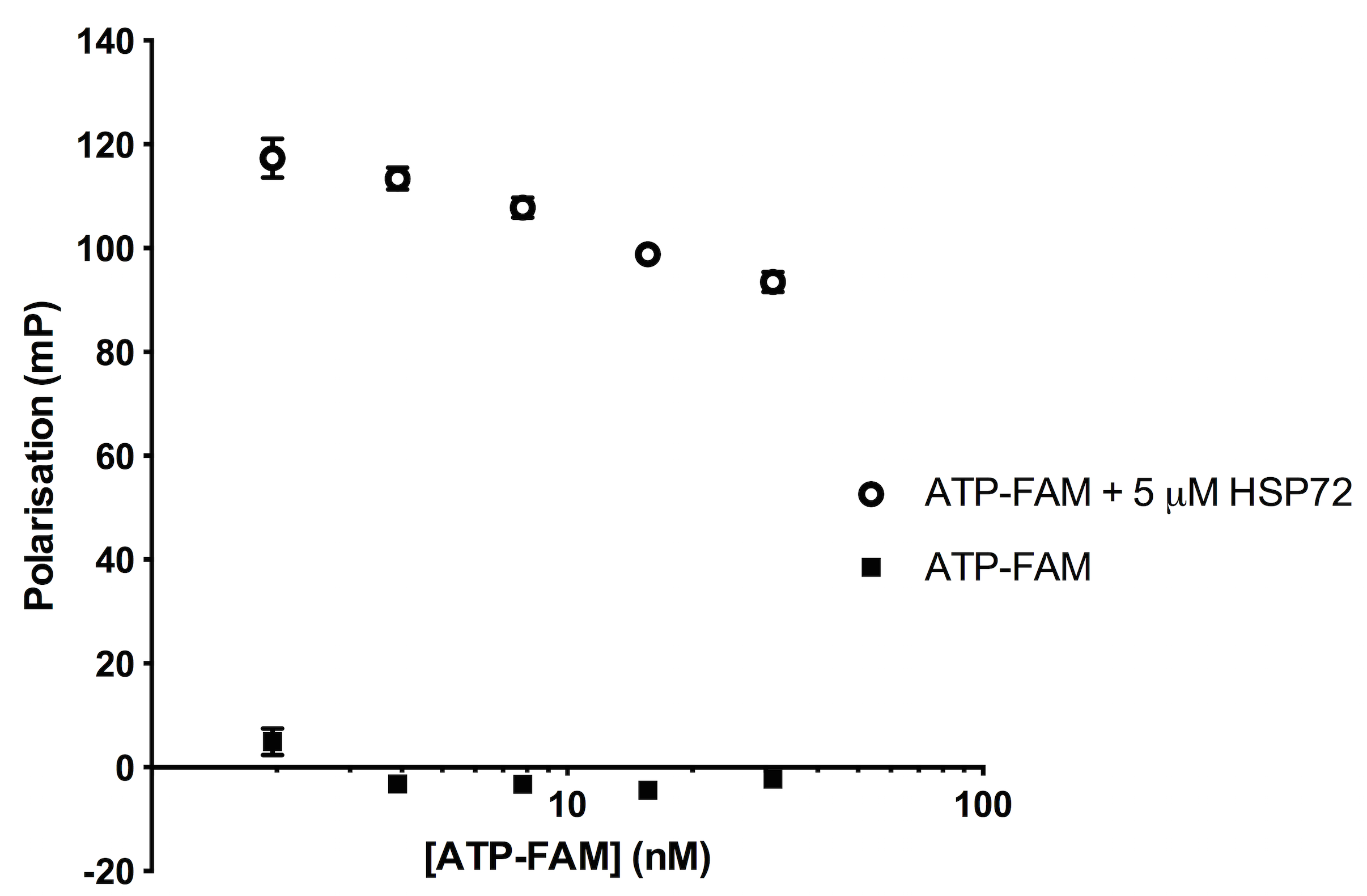

Supplement: S1 Fig — Polarization values (mP) for 2–31 nM ATP-FAM in the presence and absence of 5 μM HSP72. Assay was performed in triplicate and the mean and standard error plotted using GraphPad Prism 6. (TIFF) [file pone.0140006.s001.tiff]

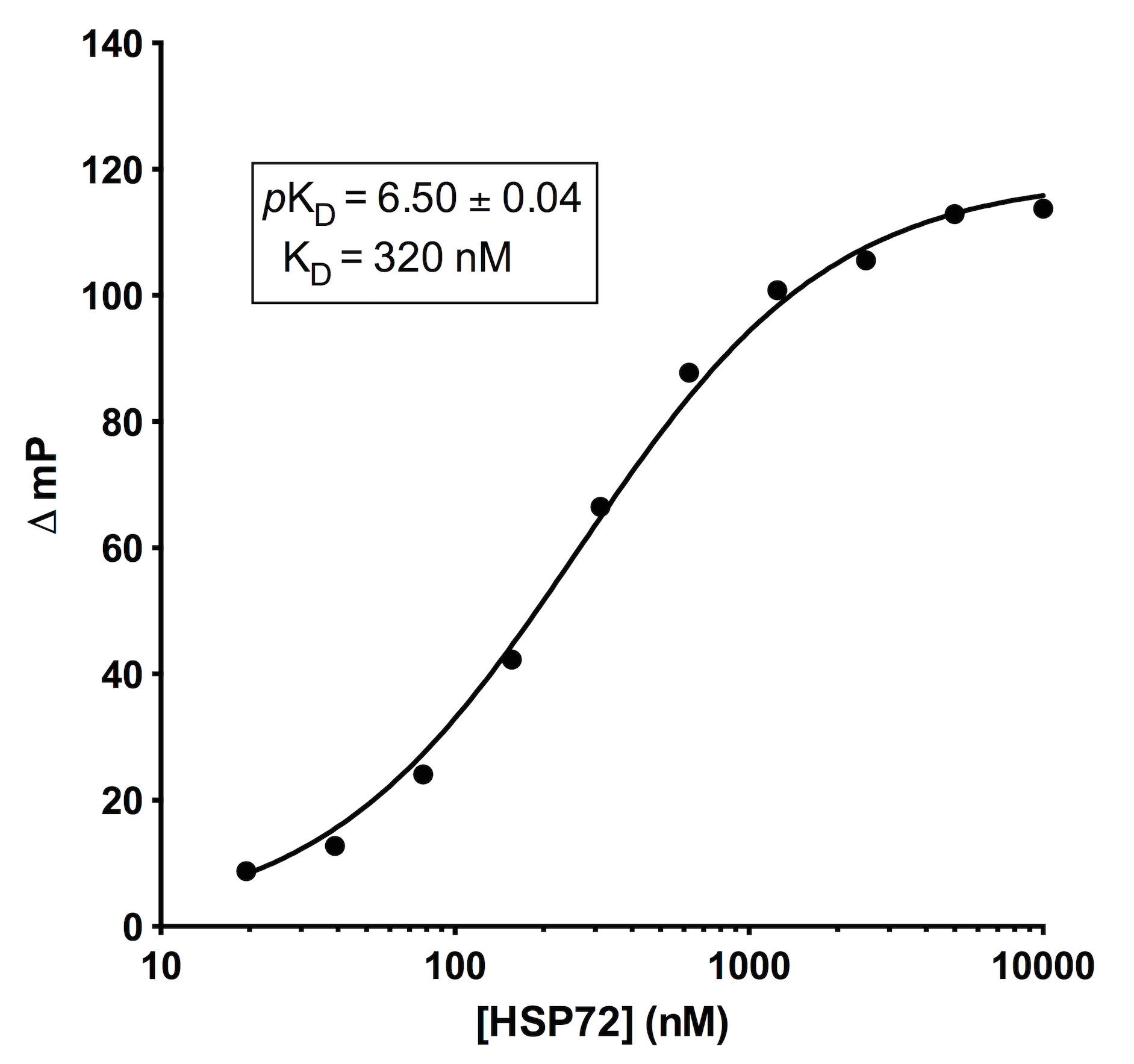

Supplement: S2 Fig — Assay was performed in triplicate and the mean and standard error plotted using GraphPad Prism 6. KD value is the geometric mean and pKD values are the geometric mean ± SE from 3 independent measurements. (TIFF) [file pone.0140006.s002.tiff]

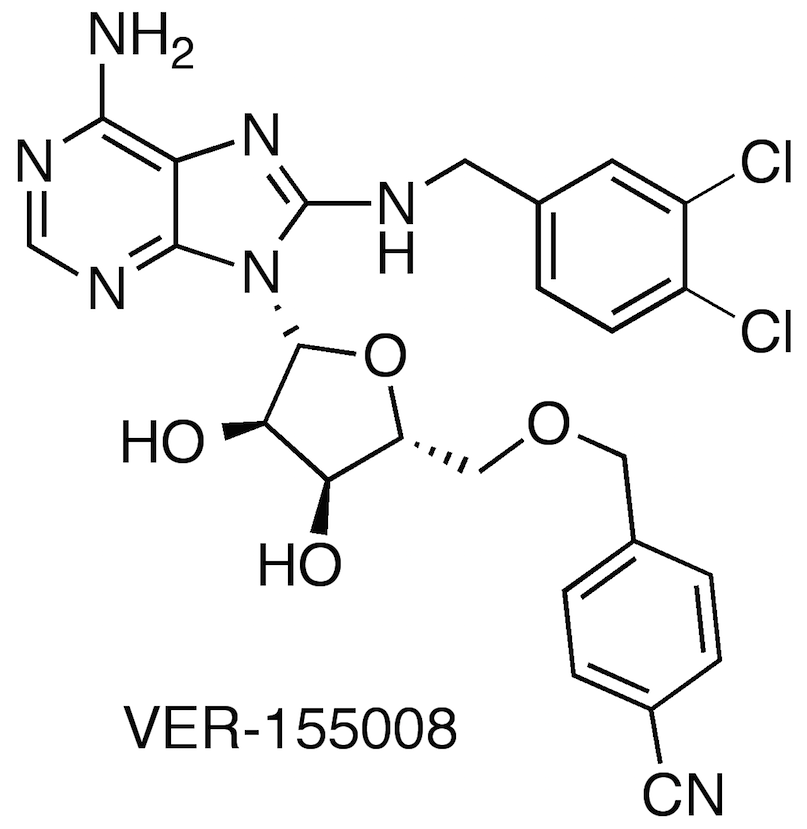

Supplement: S3 Fig — (TIF) [file pone.0140006.s003.tif]

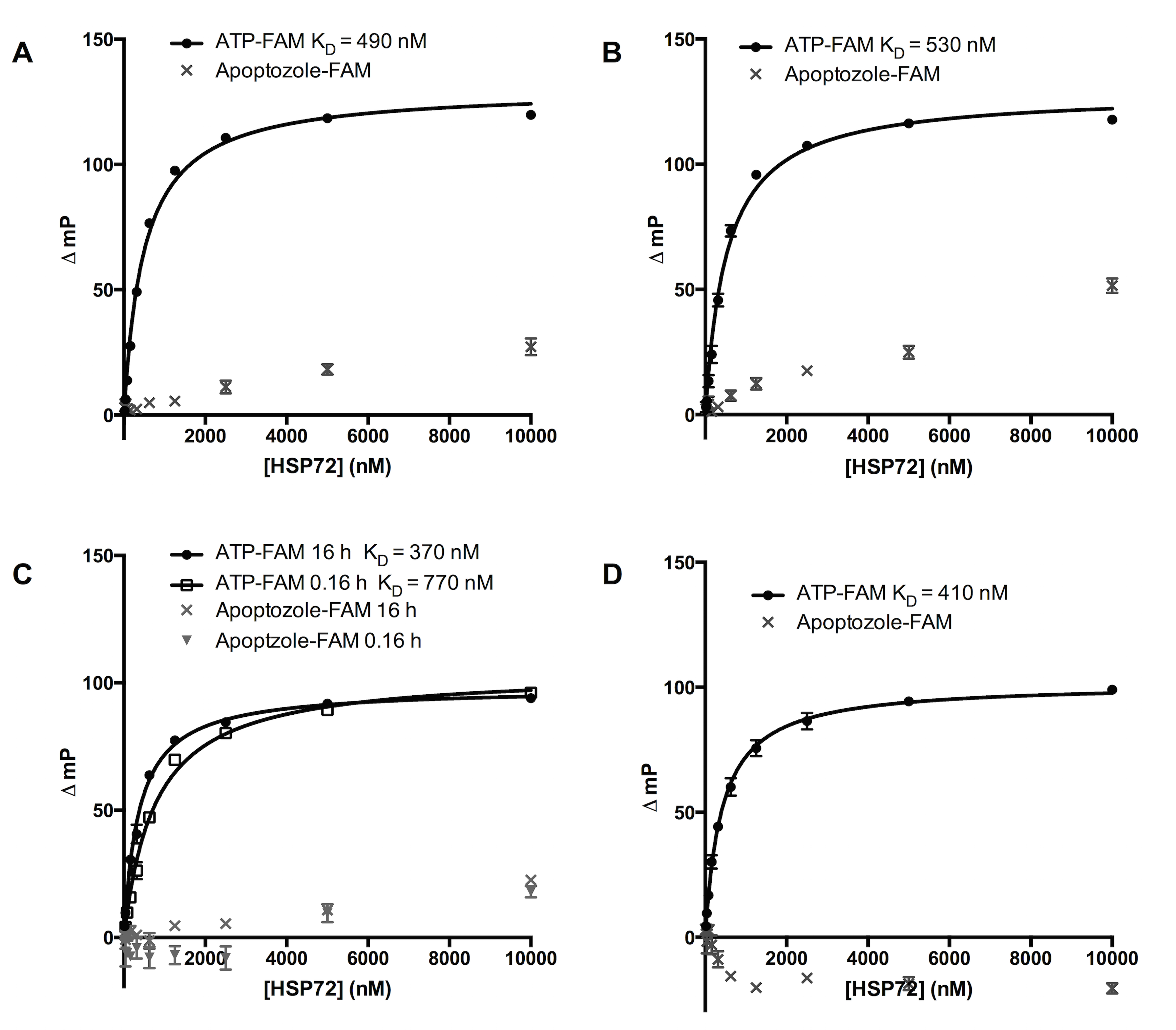

Supplement: S4 Fig — Binding interaction not observed under any of the assay conditions tested, KD values are for a single determination. (TIFF) [file pone.0140006.s004.tiff]

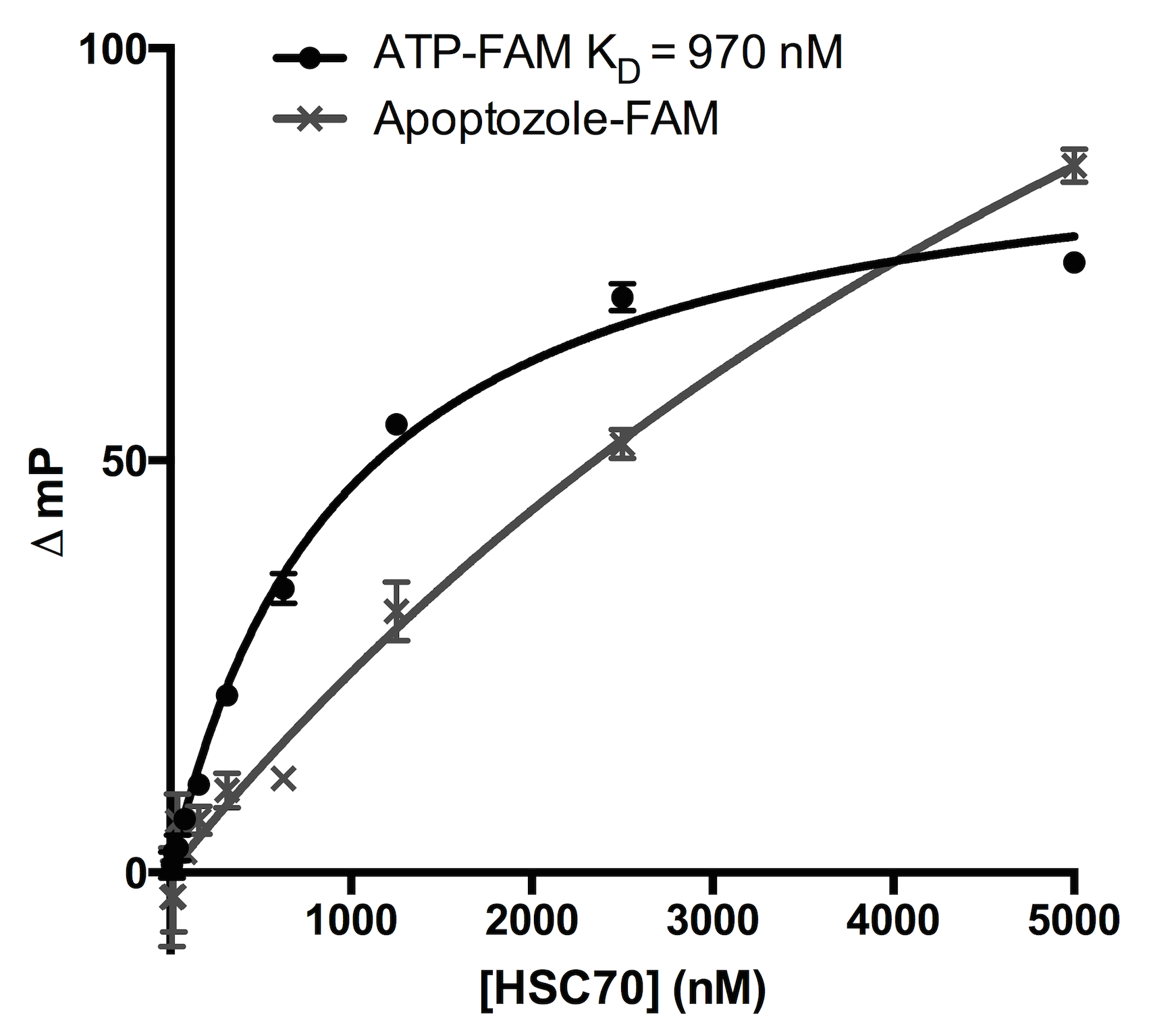

Supplement: S5 Fig — KD values are for a single determination. (TIFF) [file pone.0140006.s005.tiff]

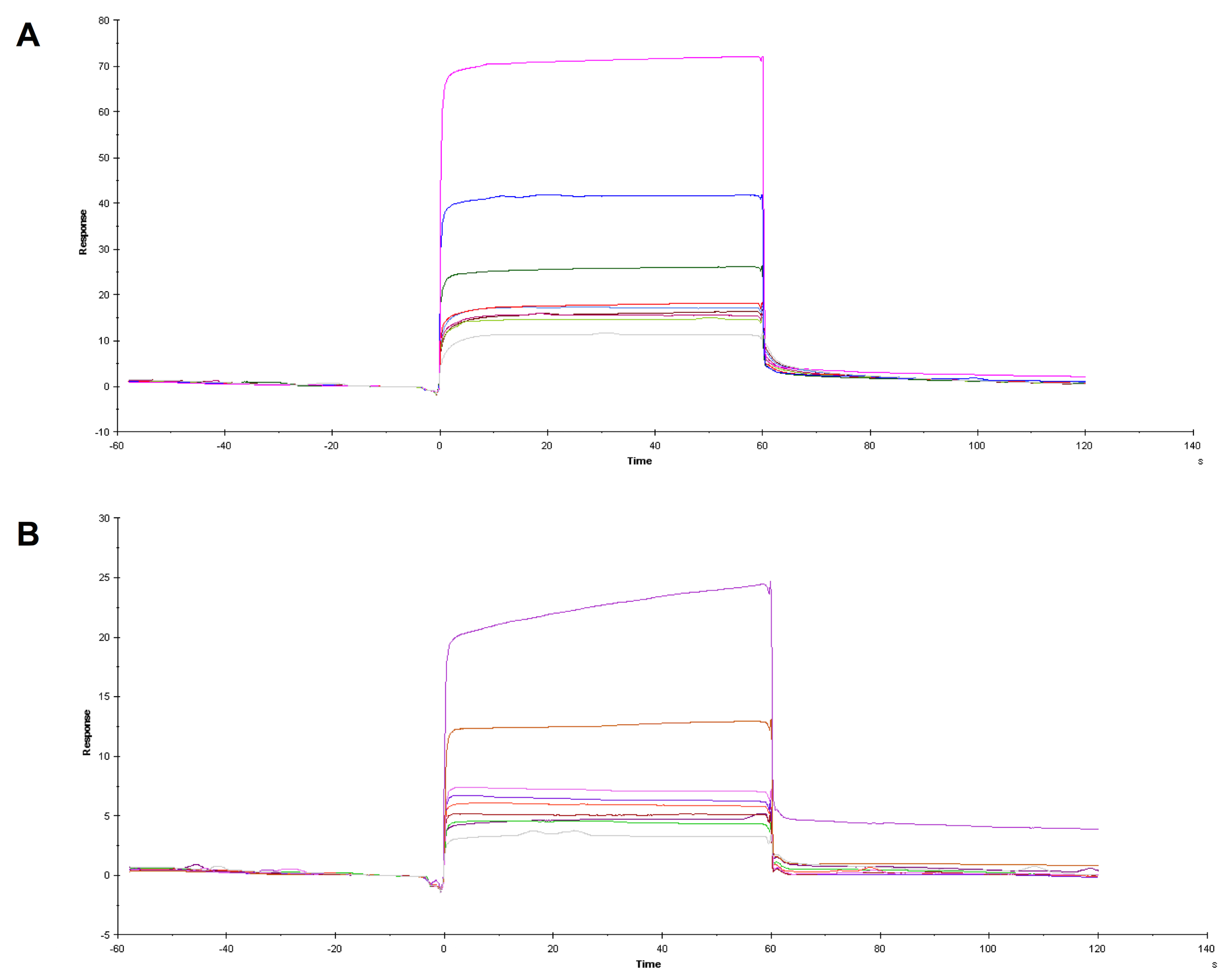

Supplement: S6 Fig — Biotinylated apoptozole 4 was immobilized on a neutravidin-derivatised gold chip followed by injection of HSP72 (31–500 nM), individual protein concentrations are highlighted by colored traces. (TIFF) [file pone.0140006.s006.tiff]
