# Supplementary material for: Investigating Apoptozole as a Chemical Probe for HSP70 Inhibition
Source: PLoS One. 2015 Oct 12;10(10):e0140006. doi: 10.1371/journal.pone.0140006 (PMC4601772; doi:10.1371/journal.pone.0140006)
Supplement: S1 Appendix — (DOCX) [file pone.0140006.s007.docx]

**Supporting information**

**Investigating Apoptozole as a Chemical Probe for HSP70 Inhibition**

Lindsay E. Evans, Matthew D. Cheeseman, Norhakim Yahya and Keith Jones*

**Corresponding Author:** keith.jones@icr.ac.uk


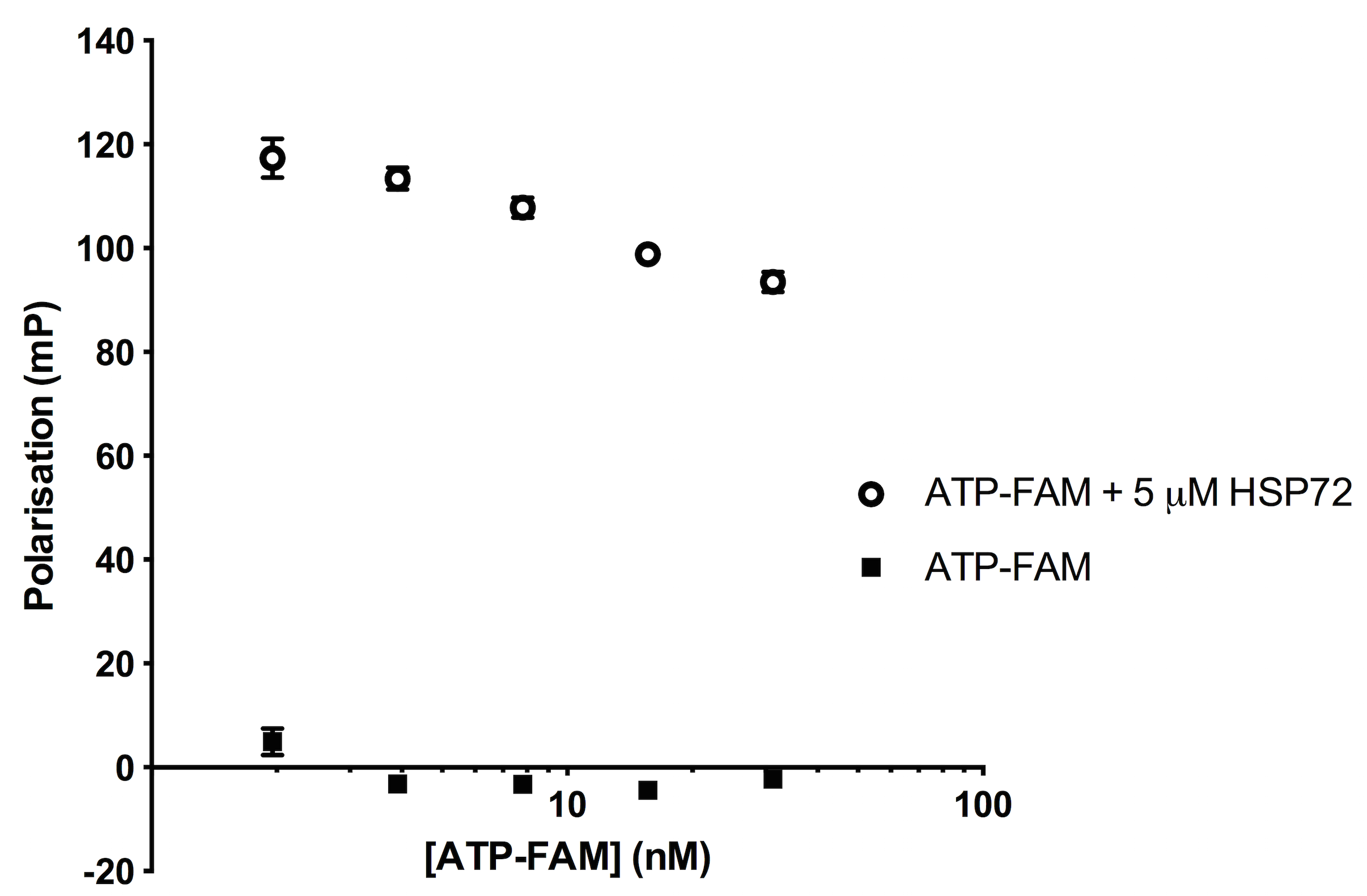


**S1 Fig. Determination of assay binding window for ATP-FAM.** Polarization values (mP) for 2-31 nM ATP-FAM in the presence and absence of 5 μM HSP72.


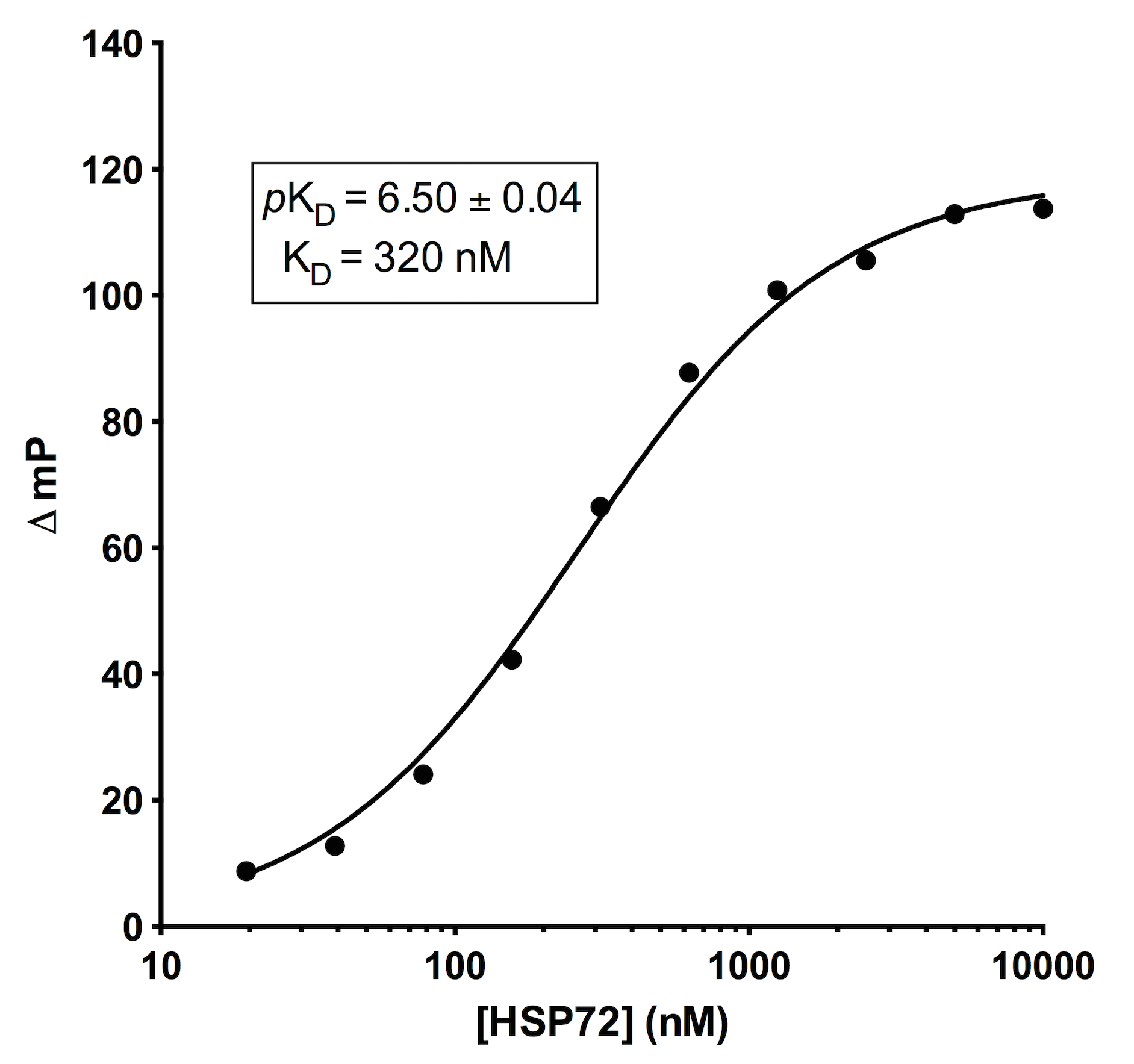


**S2 Fig. Binding isotherm for HSP72 and 10 nM ATP-FAM.** K_D_ value is the geometric mean and *p*K_D_ values are the geometric mean ± SE from 3 independent measurements.


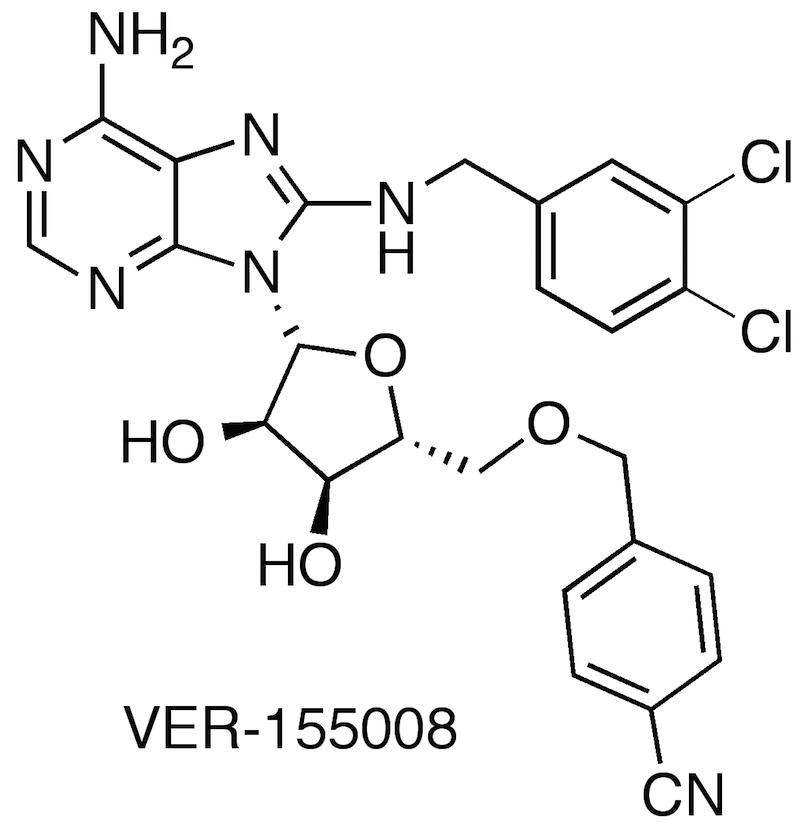


**S3 Fig. Chemical structure of ATP-competitive HSP70 inhibitor VER-155008.**


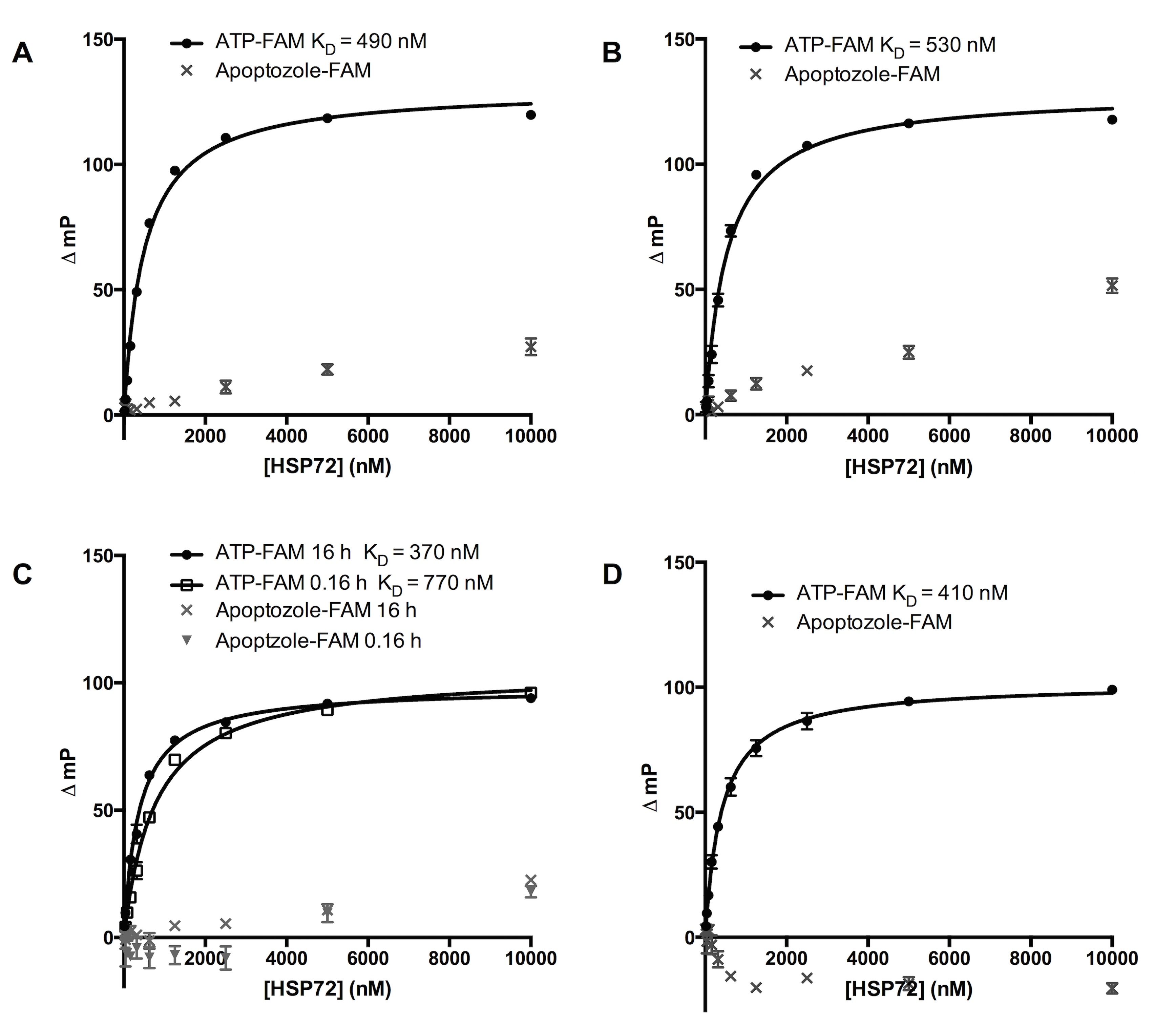


**S4 Fig. Change in metal ions, detergent and length of incubation has no effect on binding of apoptozole-FAM to HSP72.** Binding interaction not observed under any of the assay conditions tested, K_D_ values are for a single determination. (A) K_D_ determination with 0.005% Tween 20; (B) K_D_ determination with 0.01% Triton X-100; (C) K_D_ determination after 0.16 and 16 hours incubation; (D) K_D_ determination with buffer conatining 150 nM KCl and 5 nM CaCl_2_.


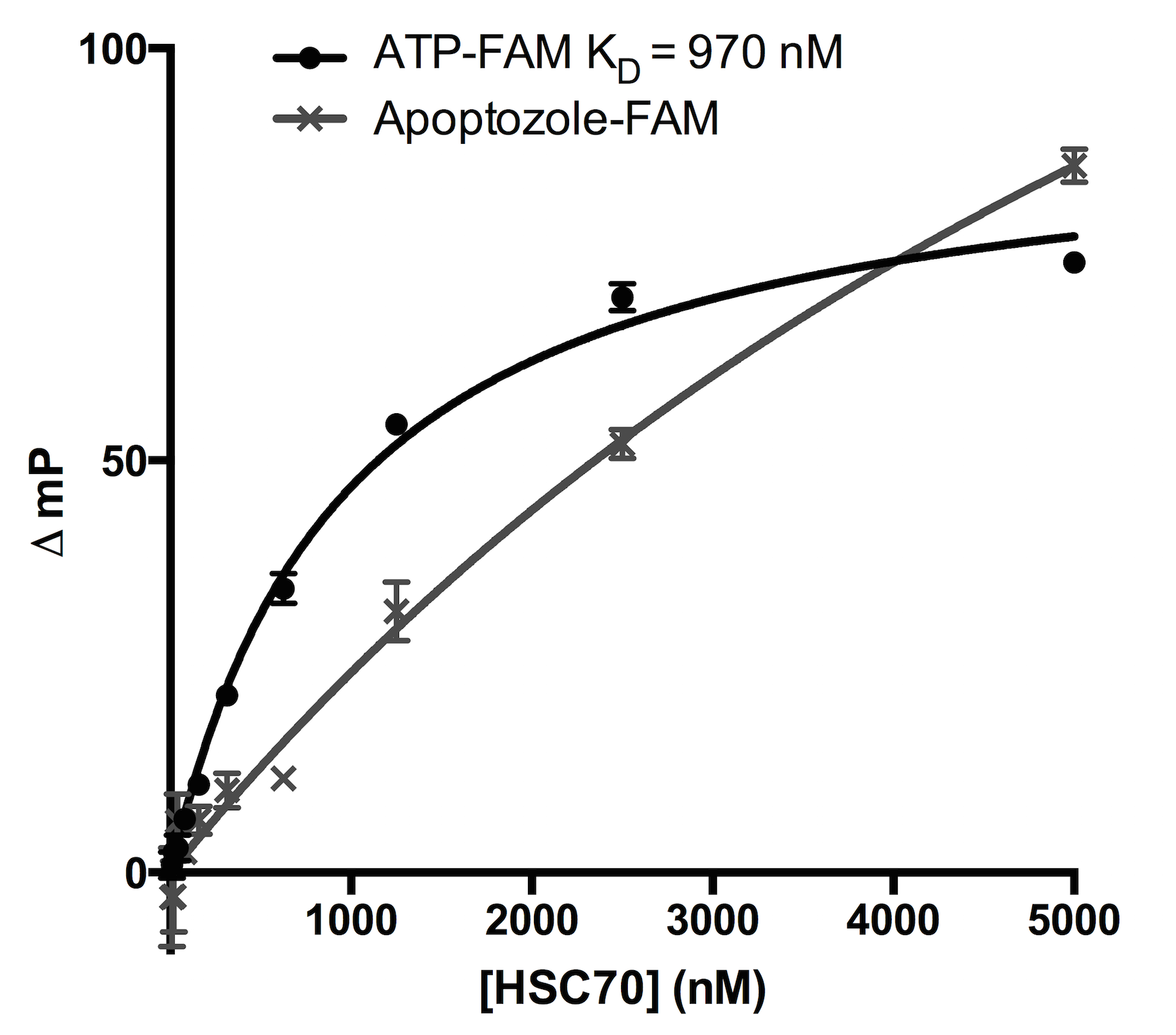


**S5 Fig. Apoptozole-FAM does not bind to HSC70.** K_D_ determination with human HSC70, K_D_ values are for a single determination.


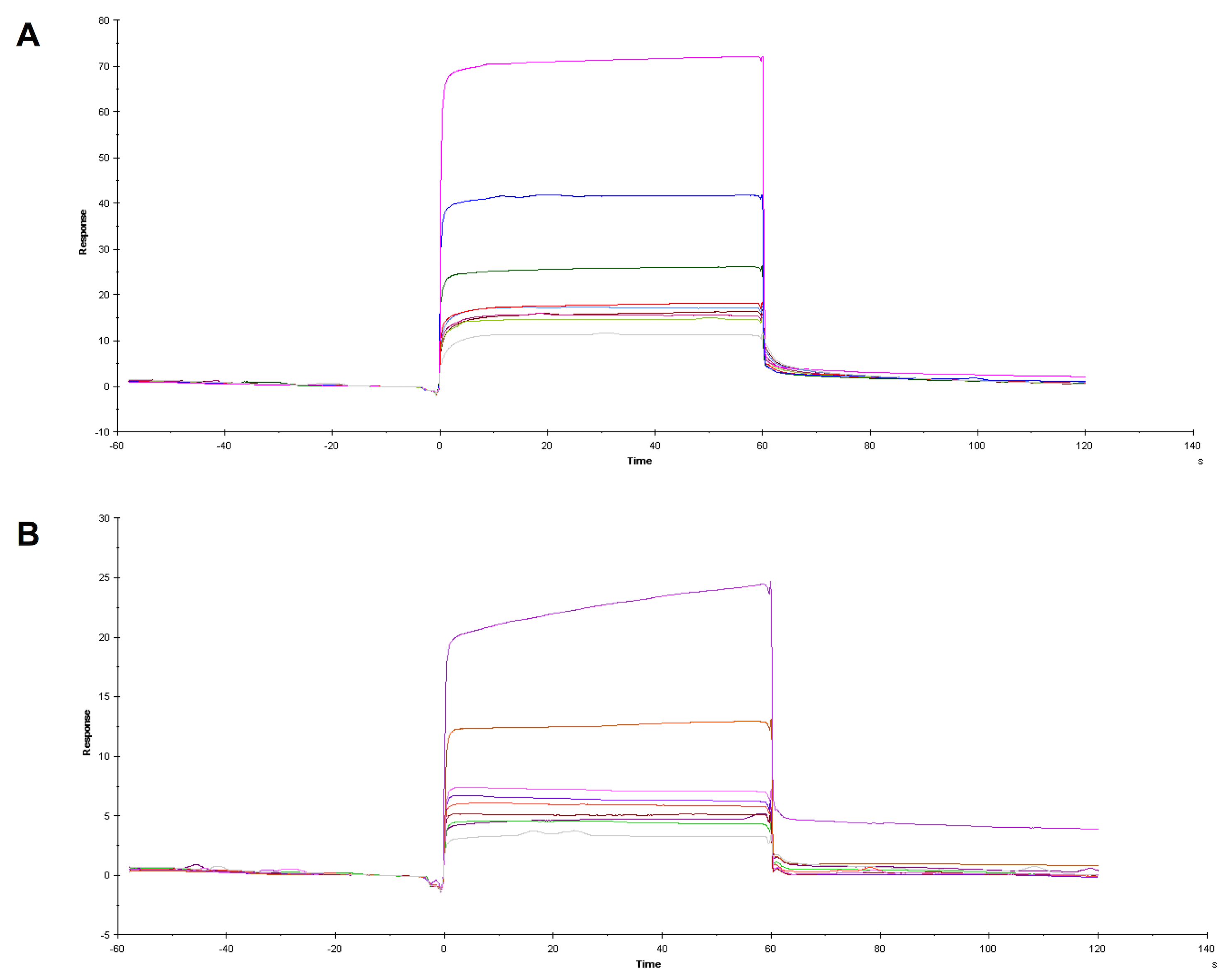


**S6 Fig. Surface Plasmon Resonance analysis of HSP72 binding to apoptozole prior to subtraction of control lane.** Biotinylated apoptozole **4** was immobilized on a neutravidin-derivatised gold chip followed by injection of HSP72 (31 - 500 nM), individual protein concentrations are highlighted by colored traces. (A) Rat HSP72 sensorgram prior to subtraction of control lane data; (B) Human HSP72 sensorgram prior to subtraction of control lane data.

**S1 Appendix. Chemical Synthesis**

Reagents and solvents were purchased from commercial suppliers (Acros, Alfa Aesar, Apollo, Cambridge peptides, Fisher scientific, Fluorochem, Lumiprobe, Manchester Organics, Sigma-Aldrich, Thermo Scientific and VWR) and used without further purification. *N*-Boc-3,6-dioxa-1,8-octanediamine[[1](#_ENREF_1)] and biotin-derivative **7**[[2](#_ENREF_2)] were prepared according to literature procedure.


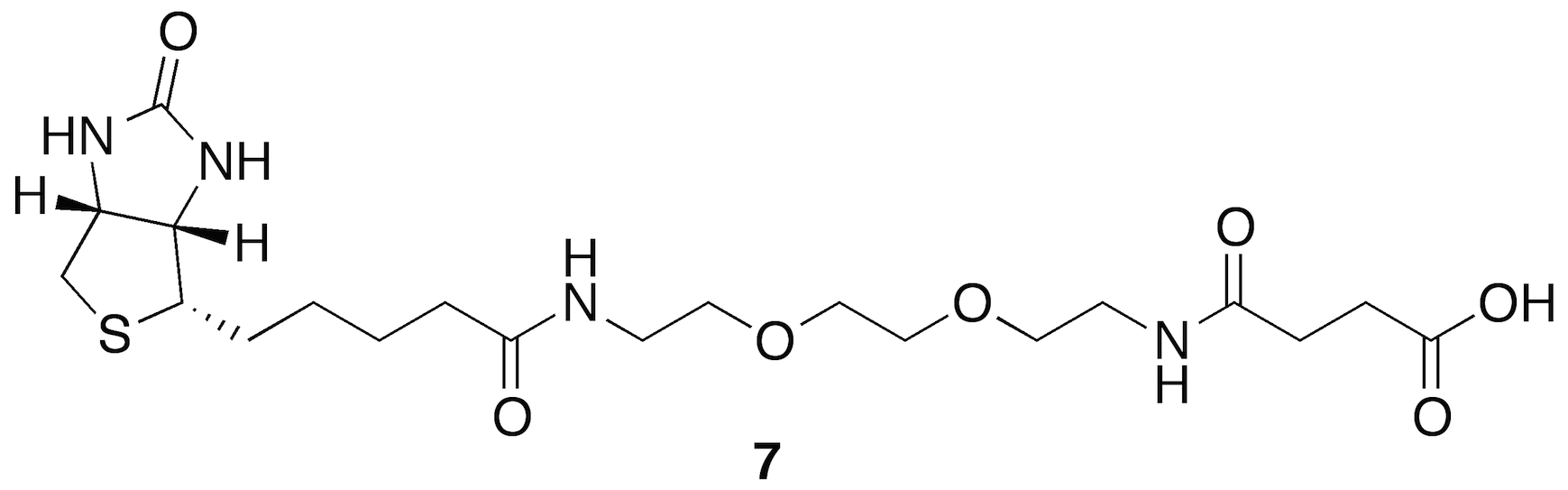


Analytical thin layer chromatography (TLC) was performed on pre-coated aluminium sheets (60 F245 nm, Merck) and visualised by short-wave UV light and potassium permanganate dips. Flash column chromatography was carried out using Merck silica gel 60 (40-65 µm).

Semi-preparative HPLC separation was carried out at room temperature using Gilson GX-281 Liquid Handler system combined with a Gilson 322 HPLC pump (Gilson, Middleton, USA) over a 15 minute gradient elution (Grad15mins20mlsLipo.m) from 40:60 to 100:0 methanol:water (both modified with 0.1% formic acid) at a flow rate of 20 mL/min. UV-Vis spectra were acquired at 254nm on a Gilson 156 UV-Vis detector (Gilson, Middleton, USA). Collection was triggered by UV signal, and collected using a Gilson GX-281 Liquid Handler system (Gilson, Middleton, USA).

^1^H NMR spectra were recorded on a Bruker AMX500 (500 MHz) spectrometer using an internal deuterium lock. Chemical shifts were measured in parts per million (ppm) relative to tetramethylsilane (*δ* = 0) using the following residual solvent signals: CDCl_3_ (δ_H_ 7.26), CD_3_OD (δ_H_ 3.32), and (CD_3_)_2_SO (δ_H_ 2.50). Multiplicities are recorded as singlet (s), doublet (d), triplet (t), quartet (q) and multiplet (m), doublet of doublets (dd), doublet of doublet of doublets (ddd), apparent (app), obscured (obs) and broad (br). Coupling constants, *J*, are measured to the nearest 0.1 Hz. ^13^C NMR spectra were recorded at 126 MHz on a Bruker Avance 500 MHz spectrometer using an internal deuterium lock. Chemical shifts were measured in parts per million (ppm) relative to tetramethylsilane (*δ* = 0) using the following residual solvent signals: CHCl_3_ (δ_C_ 77.16), CD_3_OD (δ_c_ 49.00) and (CD_3_)_2_SO (δ_C_ 39.52). Chemical shifts are quoted to 0.1 ppm, unless greater accuracy is required.

High resolution mass spectrometry was performed on an Agilent 1200 series HPLC and diode array detector coupled to a 6210 time of flight mass spectrometer with dual multimode APCI/ESI source. Analytical separation was carried out at 30 °C on a Merck Chromolith SpeedROD column (RP-18e, 50 x 4.6 mm) or a Merck Purospher STAR column (RP-18e, 30 x 4 mm) using a flow rate of 1.5 mL/min in a 4 minute gradient elution; solvents – aqueous (0.1% formic acid) and methanol. UV Detection was at 254 nm. LC/MS analysis was performed on a Waters Alliance 2795 separations module and a Waters 2487 dual wavelength absorbance detector coupled to a Waters/Micromass LCt time of flight mass spectrometer with ESI source. Analytical separation was carried out at 30°C on a Merck Purospher STAR column (RP-18e, 30 x 4 mm) using a flow rate of 1.5 mL/min in a 4 minute gradient elution with detection at 254 nm.

**Apoptozole**

**
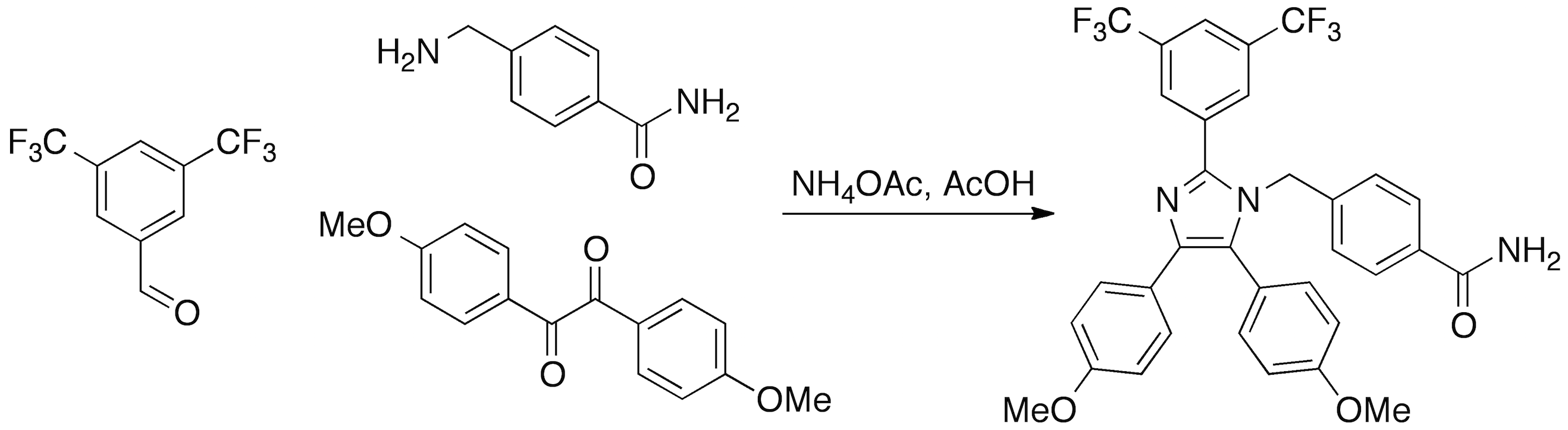
**

Apoptozole was prepared by slight modification of a literature procedure.[[3](#_ENREF_3)] 4-(Aminomethyl) benzamide (0.14 g, 0.96 mmol), 3,5-bis(trifluoromethyl)benzaldehyde (0.16 mL, 0.96 mmol), 1,2-bis(4-methoxyphenyl)ethane-1,2-dione (0.2 g, 0.74 mmol) and ammonium acetate (0.34 g, 4.44 mmol) were added to acetic acid (6 mL) and stirred at 100 °C for 16 hours. The reaction was cooled to room temperature, diluted with EtOAc and washed with water, saturated aqueous NaHCO_3_ solution and brine. The organic layer was dried over MgSO_4_ and the solvent removed under reduced pressure to give the crude product which was purified by column chromatography (9:1 CH_2_Cl_2_/MeOH; R_f_ 0.42) to give **apoptozole** as a white solid (0.21 g, 45%); mp 207.6 – 211.0 °C; δ_H_ (500 MHz, CD_3_OD) 8.17 (s, 2H), 8.01 (s, 1H), 7.75 (d, *J* = 8.5 Hz, 2H), 7.41 (d, *J* = 9.0 Hz, 2H), 7.26 (d, *J* = 8.8 Hz, 2H), 7.00 – 6.92 (m, 4H), 6.82 (d, *J* = 9.0 Hz, 2H), 5.27 (s, 2H), 3.81 (s, 3H), 3.76 (s, 3H); δ_C_ (126 MHz, CD_3_OD) 171.5, 161.9, 160.4, 145.8, 142.3, 140.0, 134.4, 134.2, 133.6, 133.2 (app d, *J* = 33.6 Hz), 132.3, 130.28 (app d, *J* = 2.3 Hz), 129.5, 129.3, 127.5, 127.1, 123.6, 123.0, 115.6, 114.7, 55.80, 55.7, 49.3; HRMS (ESI) C_33_H_26_F_6_N_3_O_3_ (M+H^+^) requires 626.1873, found 626.1852.

**4-((2-(3,5-Bis(trifluoromethyl)phenyl)-4,5-bis(4-methoxyphenyl)-1*H*-imidazol-1-yl)methyl)benzoic acid (1)**

**
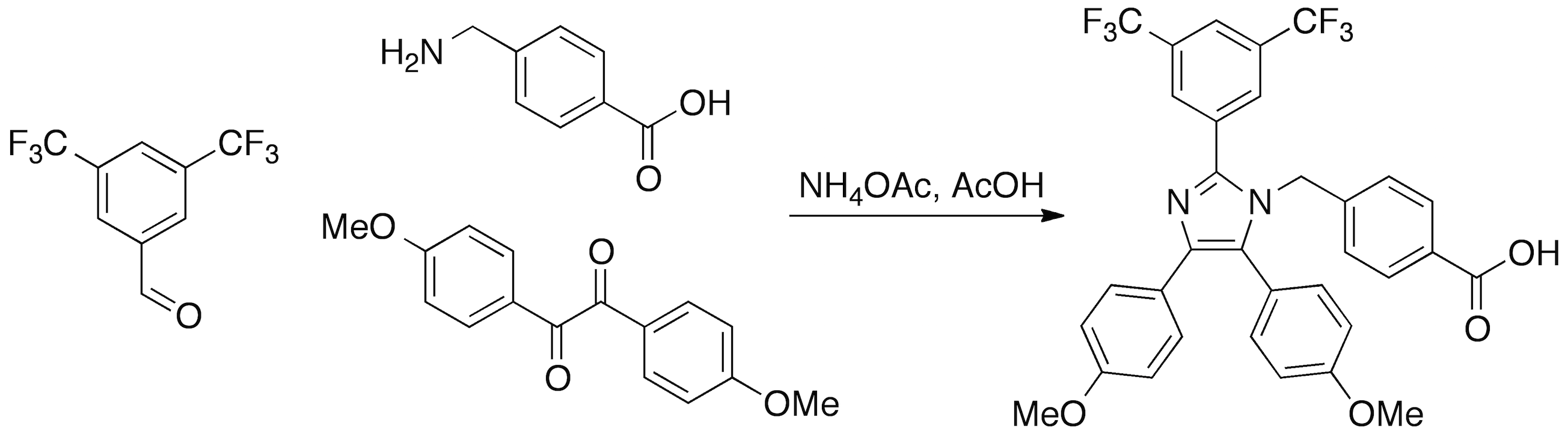
**

Compound **1** was prepared by slight modification of a literature procedure.[[3](#_ENREF_3)] 3,5-Bis(trifluoromethyl)benzaldehyde (0.48 mL, 2.89 mmol), ammonium acetate (1.03 g, 13.32 mmol), 4-(aminomethyl)benzoic acid (0.44 g, 2.89 mmol) and 1,2-bis(4-methoxyphenyl)ethane-1,2-dione (0.60 g, 2.22 mmol) were added to acetic acid (18.5 mL) and stirred at 100 °C for 16 hours. The reaction was cooled to room temperature, diluted with EtOAc and washed with water, saturated aqueous NaHCO_3_ solution and brine. The organic layer was dried over MgSO_4_ and the solvent removed under reduced pressure to give the crude product which was purified by column chromatography (2:3 cyclohexane/CH_2_Cl_2_, 0.1% AcOH; R_f_ 0.24) to give the title compound (**1**) as a white solid (0.57 g, 41%); mp 215.0 – 218.7 °C; δ_H_ (500 MHz, CDCl_3_) 8.06 (s, 2H), 7.99 (d, *J* = 8.3 Hz, 2H), 7.83 (s, 1H), 7.51 (d, *J* = 8.9 Hz, 2H), 7.18 (d, *J* = 8.7 Hz, 2H), 6.98 (d, *J* = 8.3 Hz, 2H), 6.89 (d, *J* = 8.8 Hz, 2H), 6.80 (d, *J* = 8.9 Hz, 2H), 5.15 (s, 2H), 3.82 (s, 3H), 3.77 (s, 3H), 2.12 (s, 1H); δ_C_ (126 MHz, (CD_3_)_2_SO) 166.8, 159.7, 158.1, 143.3, 141.9, 137.6, 133.0, 132.2, 130.61, 130.59 (q, *J* = 33.1 Hz), 129.7, 128.4 (app d, *J* = 2.4 Hz) 127.4, 126.6, 125.9, 124.1, 122.0, 121.8, 114.6, 113.6, 55.1, 55.0, 47.7; HRMS (ESI) C_33_H_25_F_6_N_2_O_4_ (M+H^+^) requires 627.1713, found 627.1707.

***tert*-Butyl(2-(2-(2-(4-((2-(3,5-bis(trifluoromethyl)phenyl)-4,5-bis(4-methoxyphenyl)-1*H*-imidazol-1-yl)methyl)benzamido)ethoxy)ethoxy) ethyl)carbamate (2)**


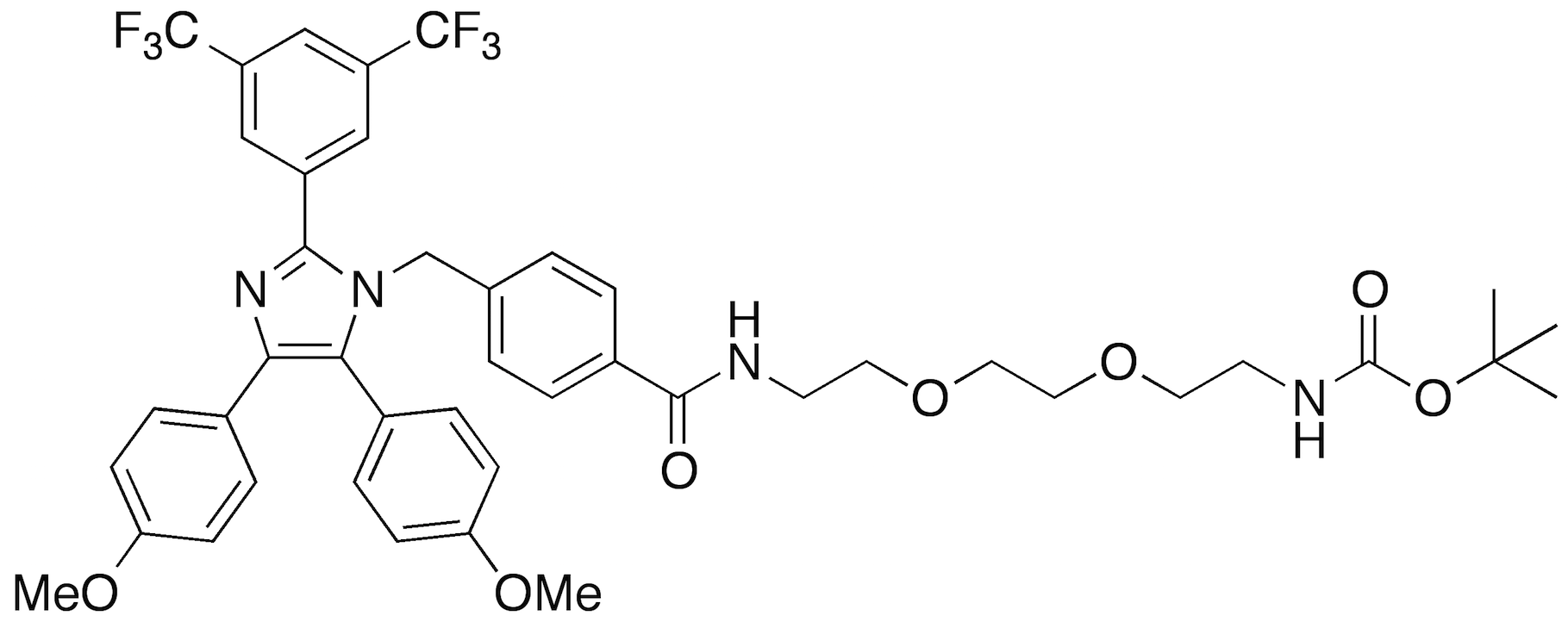


*N*-Boc-3,6-dioxa-1,8-octanediamine (59 mg, 0.24 mmol) was dissolved in CH_2_Cl_2_ (1 mL) and added to a stirred solution of **1** (0.10 g, 0.16 mmol) in CH_2_Cl_2_ (5 mL). HBTU (70 mg, 0.19 mmol) and DIPEA (84 μL, 0.48 mmol) were added and the reaction stirred at room temperature for 5 hours. The reaction mixture was concentrated under reduced pressure and the crude product purified by column chromatography (2% MeOH in CH_2_Cl_2_; R_f_ 0.3) to give the title compound (**2**) as colourless oil (80 mg, 59%); δ_H_ (500 MHz, CDCl_3_) 8.07 (s, 2H), 7.83 (s, 1H), 7.72 (d, *J* = 7.8 Hz, 2H), 7.51 (d, *J* = 8.8 Hz, 2H), 7.17 (d, *J* = 8.1 Hz, 2H), 6.95 (d, *J* = 7.7 Hz, 2H), 6.89 (d, *J* = 8.6 Hz, 2H), 6.79 (d, *J* = 8.9 Hz, 2H), 6.70 (br s, 1H), 5.13 (s, 2H), 4.98 (br s, 1H), 3.82 (s, 3H), 3.77 (s, 3H), 3.69 – 3.61 (m, 8H), 3.56 – 3.50 (m, 2H), 3.32 – 3.25 (m, 2H), 1.40 (s, 9H); δ_C_ (126 MHz, CDCl_3_) 166.7, 165.9, 160.3, 158.8, 156.1, 144.2, 134.3, 132.4, 132.1 (q, *J* = 33.7Hz) 130.5, 128.7, 128.1, 127.9, 126.4, 125.9, 124.2, 122.4, 122.0, 114.8, 113.9, 79.5, 70.4, 70.3, 70.2, 69.9, 55.4, 55.3, 48.3, 40.4, 39.9, 28.5; HRMS (ESI) C_44_H_47_F_6_N_4_O_7_ (M+H^+^) requires 857.3343, found 857.3366.

***N*-(2-(2-(2-Aminoethoxy)ethoxy)ethyl)-4-((2-(3,5-bis(trifluoromethyl)phenyl)-4,5-bis(4-methoxyphenyl)-1*H*-imidazol-1-yl)methyl)benzamide (3)**

**
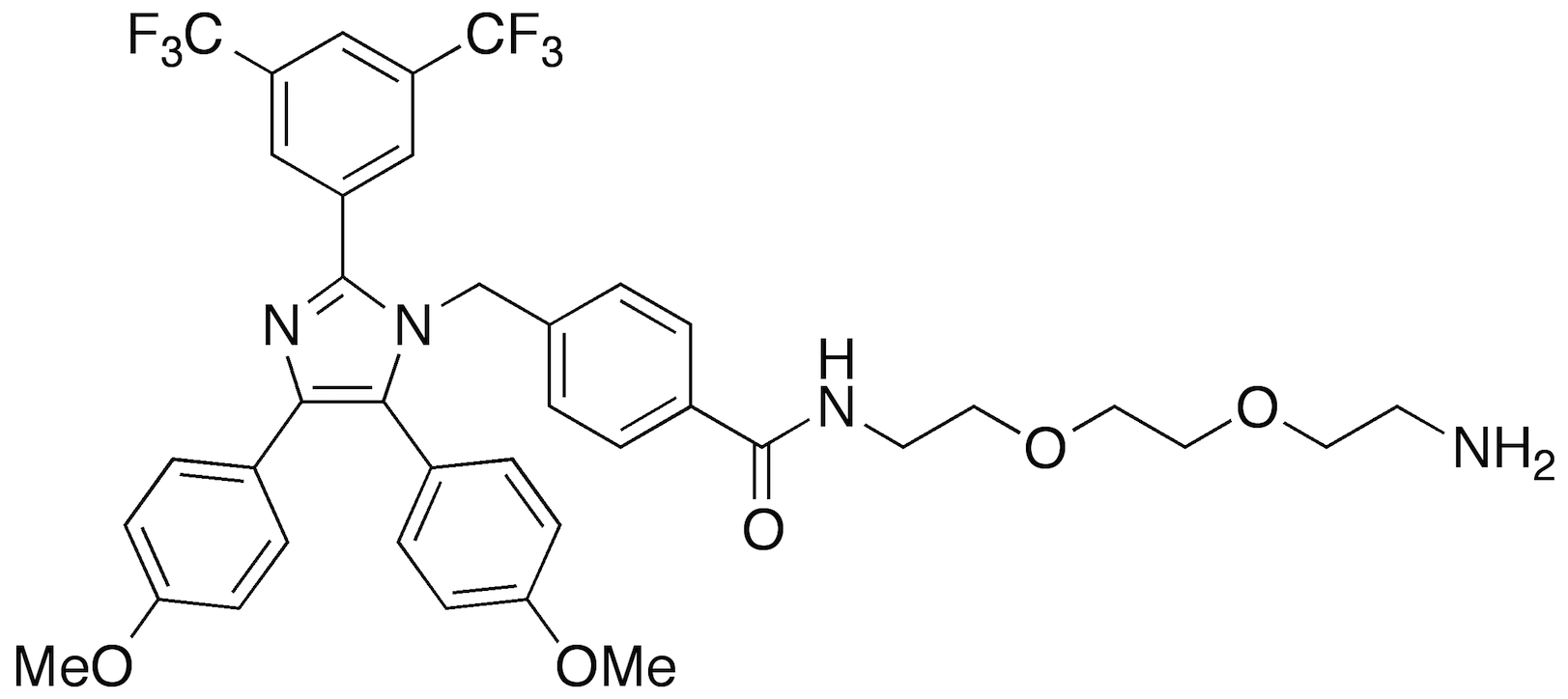
**

TFA (2 mL) was added dropwise to a solution of **2** (78 mg, 0.09 mmol) in CH_2_Cl_2_ (2 mL) and the reaction stirred at room temp for 1.5 hours. The solvent was removed under reduced pressure and the crude product purified by column chromatography (9:1 CH_2_Cl_2_/2.0 M NH3 in MeOH; R_f_ 0.78) to give to title compound (**3**) as a colorless oil (60 mg, 87%); δ_H_ (500 MHz, CD_3_OD) 8.18 (s, 2H), 8.01 (s, 1H), 7.71 (d, *J* = 8.4 Hz, 2H), 7.41 (d, *J* = 8.9 Hz, 2H), 7.25 (d, *J* = 8.8 Hz, 2H), 6.98 – 6.93 (m, 4H), 6.81 (d, *J* = 9.0 Hz, 2H), 5.27 (s, 2H), 3.81 (s, 3H), 3.76 (s, 3H), 3.67 – 3.63 (m, 6H), 3.58 – 3.53 (m, 4H), 2.88 (t, *J* = 5.2 Hz, 2H). δ_C_ (126 MHz, CD_3_OD) 169.5, 161.9, 160.4, 145.8, 142.0, 140.0, 135.0, 134.2, 133.6, 133.2 (q, *J* = 33.2 Hz), 132.34, 130.25, 129.5, 128.9, 127.51, 127.1, 123.6, 123.01, 115.6, 114.7, 71.34, 71.30, 71.2, 70.6, 55.8, 55.7, 49.2, 41.5, 40.8; HRMS (ESI) C_39_H_39_F_6_N_4_O_5_ (M+H+) requires 757.2819, found 757.2812.

**Apoptozole-FAM**


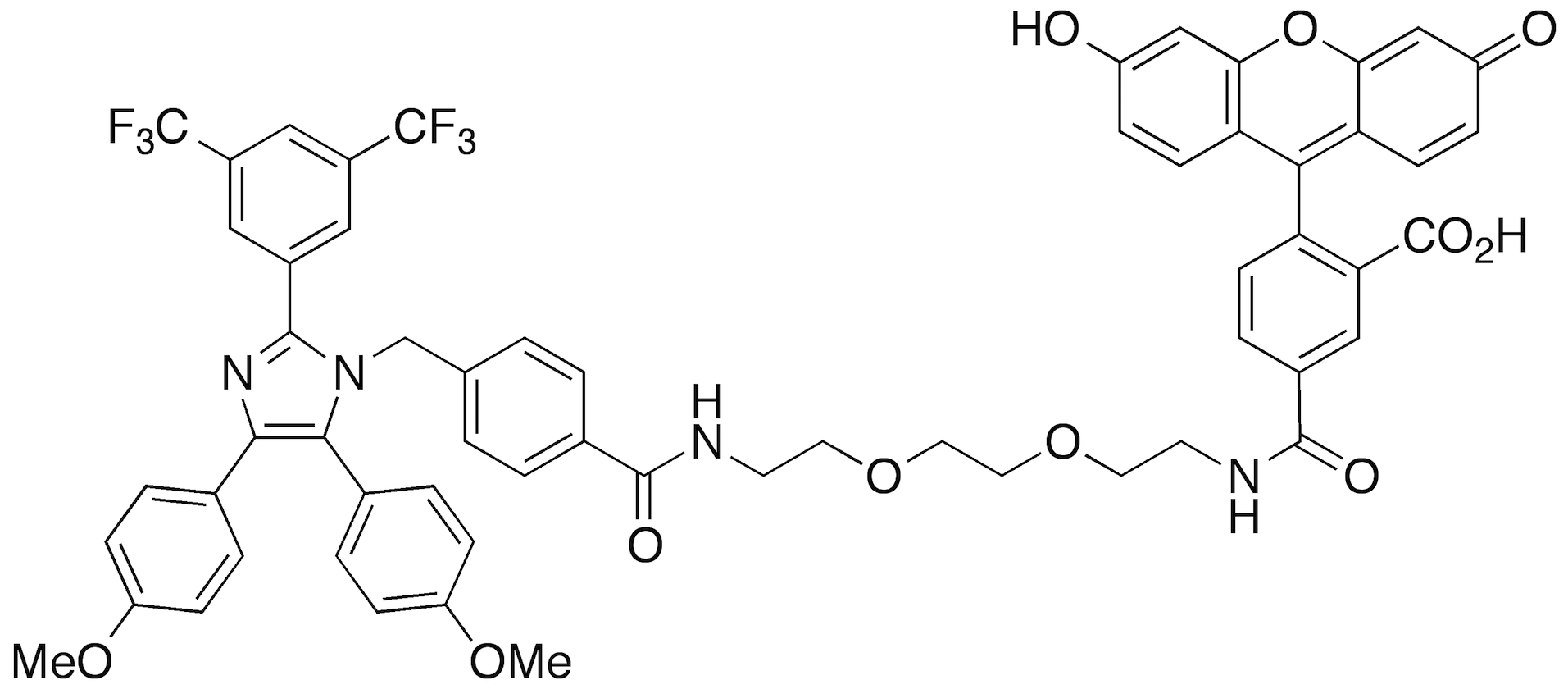


5-Carboxyfluorescein NHS ester (5 mg, 11 μmol) was dissolved in DMF (0.2 mL) and added to a stirred solution of compound **3** (9 mg, 12 μmol) in DMF (0.8 mL). Triethylamine (2 μL, 16 μmol) was added and the reaction stirred at room temperature in the dark for 16 hours. The solvent was removed under reduced pressure and the crude product purified by semi-preparative HPLC to give **apoptozole-FAM** as an orange oil (2.5 mg, 21%); δH (500 MHz, CD_3_OD) 8.41 (s, 1H), 8.18 (s, 2H), 8.09 (dd, *J* = 8.0, 1.5 Hz, 1H), 8.00 (s, 1H), 7.69 (d, *J* = 8.4 Hz, 2H), 7.39 (d, *J* = 8.9 Hz, 2H), 7.21 (d, *J* = 8.8 Hz, 2H), 7.18 (d, *J* = 8.0 Hz, 1H), 6.97 – 6.88 (m, 4H), 6.78 (d, *J* = 9.0 Hz, 2H), 6.68 (d, *J* = 2.3 Hz, 2H), 6.63 (d, *J* = 8.2 Hz, 2H), 6.52 (dd, *J* = 8.8, 2.3 Hz, 2H), 5.23 (s, 2H), 3.76 (s, 3H), 3.74 (s, 3H), 3.72 – 3.62 (m, 8H), 3.60 (t, *J* = 5.5 Hz, 2H), 3.53 (t, *J* = 5.5 Hz, 2H); δC (126 MHz, CD3OD) 161.9, 160.3, 145.8, 142.0, 140.0, 137.6, 135.0, 134.2, 133.6, 133.0, 132.3, 130.6, 130.3 (app d, *J* = 2.5 Hz), 129.5, 128.9, 127.5, 127.2, 126.6, 125.76, 125.6, 123.7, 123.0, 115.6, 114.7, 103.7, 101.4, 71.34, 70.54, 70.48, 55.8, 55.6, 49.2, 41.1, 40.9; HRMS (ESI) C60H49F6N4O11 (M+H^+^) requires 1115.3297, found 1115.3297.

**Apoptozole-Cy5**


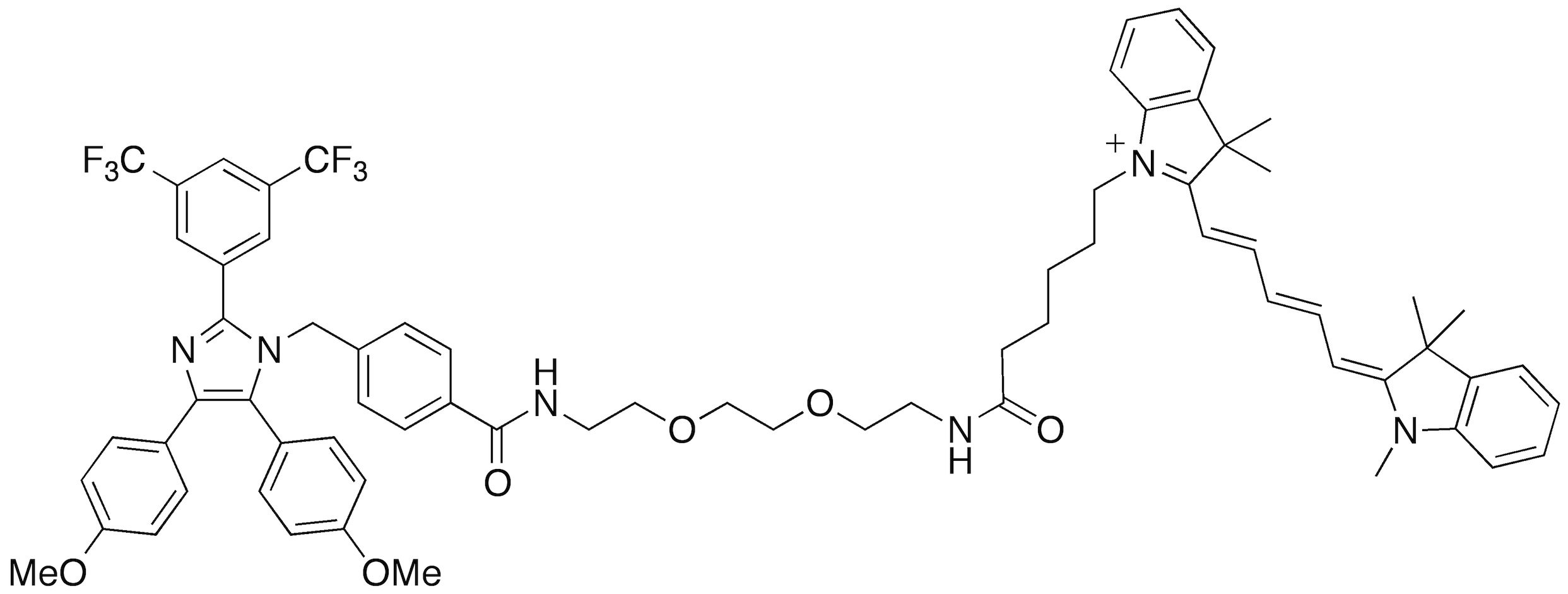


Cyanine5 NHS ester (5.0 mg, 7.9 μmol) was dissolved in DMF (0.2 mL) and added to a stirred solution of compound **3** (5.0 mg, 6.6 μmol) in DMF (0.8 mL). Triethylamine (1.5 μL, 10 μmol) was added and the reaction stirred at room temperature in the dark for 16 hours. The solvent was removed under reduced pressure and the crude product purified by semi-preparative HPLC to give **apoptozole-Cy5** as a blue oil (2.0 mg, 25%); δ_H_ (500 MHz, CDCl_3_) 8.29 (br. s, 1H), 8.16 (br. s, 1H), 8.08 (s, 2H), 8.02 (d, *J* = 8.3 Hz, 2H), 7.83 – 7.77 (m, 3H), 7.49 (d, *J* = 8.9 Hz, 2H), 7.40 – 7.31 (m, 4H), 7.21 (m, 2H), 7.16 (d, *J* = 8.7 Hz, 2H), 7.11 (d, *J* = 7.9 Hz, 1H), 7.03 (d, *J* = 8.0 Hz, 1H), 6.98 (d, *J* = 8.1 Hz, 2H), 6.90 – 6.84 (m, 3H), 6.77 (d, *J* = 8.9 Hz, 2H), 6.55 (d, *J* = 13.6 Hz, 1H), 6.26 (d, *J* = 13.4 Hz, 1H), 5.11 (s, 2H), 4.11 – 4.04 (m, 2H), 3.84 – 3.57 (m, 16H), 3.56-3.52 (m, 3H), 3.44 (app. q, *J* = 5.4 Hz, 2H), 2.32 (t, *J* = 7.3 Hz, 2H), 1.68 (s, 6H), 1.67 (s, 6H), 1.53 – 1.49 (m, 2H), 0.89 – 0.82 (m, 4H);^^[[1]](#footnote-1)^^ HRMS (ESI) C_71_H_75_F_6_N_6_O_6_^+^ (M+H^+^) requires 1221.5647, found 1221.5656.

***N*^1^-(2-(2-(2-(4-((2-(3,5-Bis(trifluoromethyl)phenyl)-4,5-bis(4-methoxyphenyl)-1*H*-imidazol-1-yl)methyl)benzamido)ethoxy)ethoxy)ethyl)-*N*^4^-(2-(2-(2-(5-((3a*S*,4*S*,6a*R*)-2-oxohexahydro-1*H*-thieno[3,4-*d*]imidazol-4-yl) pentanamido)ethoxy)ethoxy) ethyl)succinamide (4)**

**
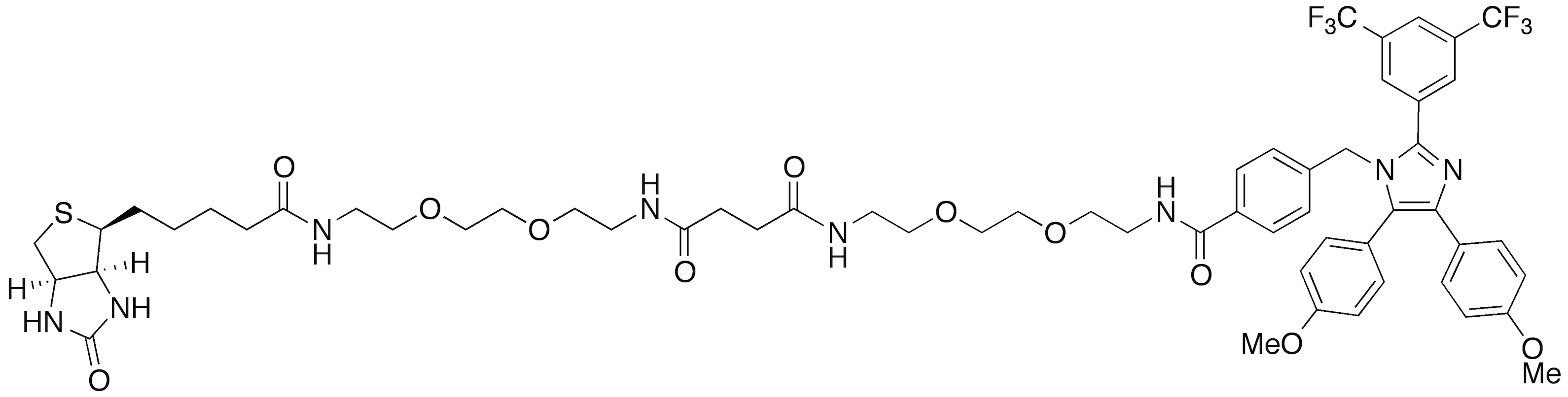
**

Biotinylated apoptozole **4** was prepared according to literature procedure.[[3](#_ENREF_3)] Compound **3** (20 mg, 26 μmol) was dissolved in DMF (2 mL) and biotin-derivative **7** (20 mg, 40 μmol), HBTU (14 mg, 40 μmol) and DIPEA (9 μL, 53 μmol) were added and the reaction stirred at room temp for 1 hour. The reaction mixture was diluted with CH_2_Cl_2_, washed with water and brine and the organic layer dried over MgSO_4_. The solvent was removed under reduced pressure and the crude product purified by semi-preparative HPLC to give the title compound (**4**) as a colorless glassy solid (11 mg, 34%); δ_H_ (500 MHz, CD_3_OD) 8.18 (s, 2H), 8.02 (s, 1H), 7.72 (d, *J* = 8.5 Hz, 2H), 7.42 (d, *J* = 9.0 Hz, 2H), 7.26 (d, *J* = 8.8 Hz, 2H), 6.96 (d, *J* = 8.8 Hz, 4H), 6.82 (d, *J* = 9.0 Hz, 2H), 5.27 (s, 2H), 4.47 (ddd, *J* = 8.0, 5.0, 1.0 Hz, 1H), 4.28 (dd, *J* = 7.9, 4.4 Hz, 1H), 3.81 (s, 3H), 3.76 (s, 3H), 3.66 – 3.58 (m, 10H), 3.56 – 3.49 (m, 8H), 3.37 – 3.32 (m, 6H), 3.18 (ddd, *J* = 8.9, 5.8, 4.4 Hz, 1H), 2.91 (dd, *J* = 12.7, 5.0 Hz, 1H), 2.69 (d, *J* = 12.7 Hz, 1H), 2.46 (s, 4H), 2.21 (t, *J* = 7.4 Hz, 2H), 1.78 – 1.52 (m, 4H), 1.48 – 1.37 (m, 2H); δ_C_ (126 MHz, CD_3_OD) 176.1, 174.7, 169.4, 161.9, 160.4, 145.8, 142.0, 140.0, 135.0, 134.2, 133.6, 133.2 (q, *J* = 33.7 Hz), 132.3, 131.7, 130.3 (app d, *J* = 3.1 Hz), 129.5, 129.0, 127.5, 127.2, 123.6, 123.0, 115.7, 114.7, 71.3, 70.62, 70.58, 70.55, 63.4, 61.6, 57.0, 55.8, 55.7, 48.0, 41.1, 40.9, 40.4, 40.3, 36.7, 32.2, 29.8, 29.5, 26.9; LCMS (ESI) *m*/*z* 607.10 (M+2H)^2+^, tr = 3.03 min.

**1-(4-((2-(3,5-Bis(trifluoromethyl)phenyl)-4,5-bis(4-methoxyphenyl)-1*H*-imidazol-1-yl)methyl)**

**phenyl)-1,12-dioxo-5,8-dioxa-2,11-diazapentadecan-15-oic acid (7)**

**
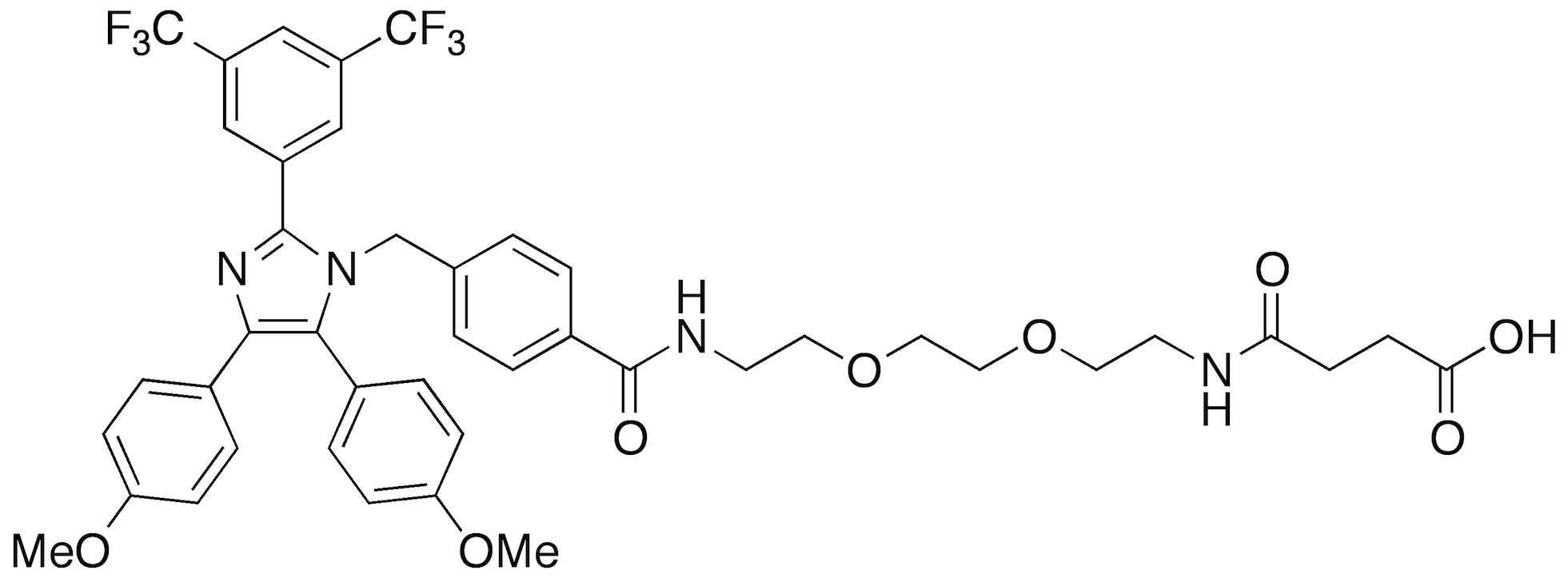
**

Compound **3** (0.17 g, 0.22 mmol) was dissolved in acetonitrile (1 mL), succinic anhydride (81 mg, 0.82 mmol) and DIPEA (0.20 ml, 1.10 mmol) were added and the reaction was stirred at 30 °C for 16 hours. The reaction was acidified with 0.1% aqueous TFA and solvent removed under reduced pressure. The crude product was purified by column chromatography (9:1 CH_2_Cl_2_/MeOH, 0.1% AcOH) to give the title compound (**7**) as a white gum (0.13 g, 68%); δ_H_ (500 MHz, CD_3_OD) 8.18 (s, 2H), 8.02 (s, 1H), 7.72 (d, *J* = 8.4 Hz, 2H), 7.41 (d, *J* = 9.0 Hz, 2H), 7.25 (d, *J* = 8.8 Hz, 2H), 6.96 (d, *J* = 8.8 Hz, 4H), 6.81 (d, *J* = 9.0 Hz, 2H), 5.27 (s, 2H), 3.81 (s, 3H), 3.76 (s, 3H), 3.66 – 3.59 (m, 8H), 3.56 – 3.50 (m, 4H), 2.53 (obs t, *J* = 7.1 Hz, 2H), 2.43 (t, *J* = 7.1 Hz, 2H); δ_C_ (126 MHz, CD_3_OD) 176.0, 174.8, 169.5, 161.9, 160.4, 145.8, 142.0, 140.0, 135.0, 134.1, 133.6, 133.2 (q, *J* = 33.8 Hz), 132.3, 130.3 (app d, *J* = 3.0 Hz), 129.5, 128.9, 127.5, 127.1, 123.6, 123.0, 115.6, 114.7, 71.3, 70.6, 70.5, 55.8, 55.7, 49.3, 40.9, 40.4, 31.5, 30.3; HRMS (ESI) C_43_H_43_F_6_N_4_O_8_ (M+2H)^2+^ requires 429.1526, found 429.1535.

***tert*-Butyl (1-(4-((2-(3,5-bis(trifluoromethyl)phenyl)-4,5-bis(4-methoxy phenyl)-1*H*-imidazol-1-yl)methyl)phenyl)-1,12,15-trioxo-5,8,19,22-tetraoxa-2,11,16-triazatetracosan-24-yl)carbamate (5)**


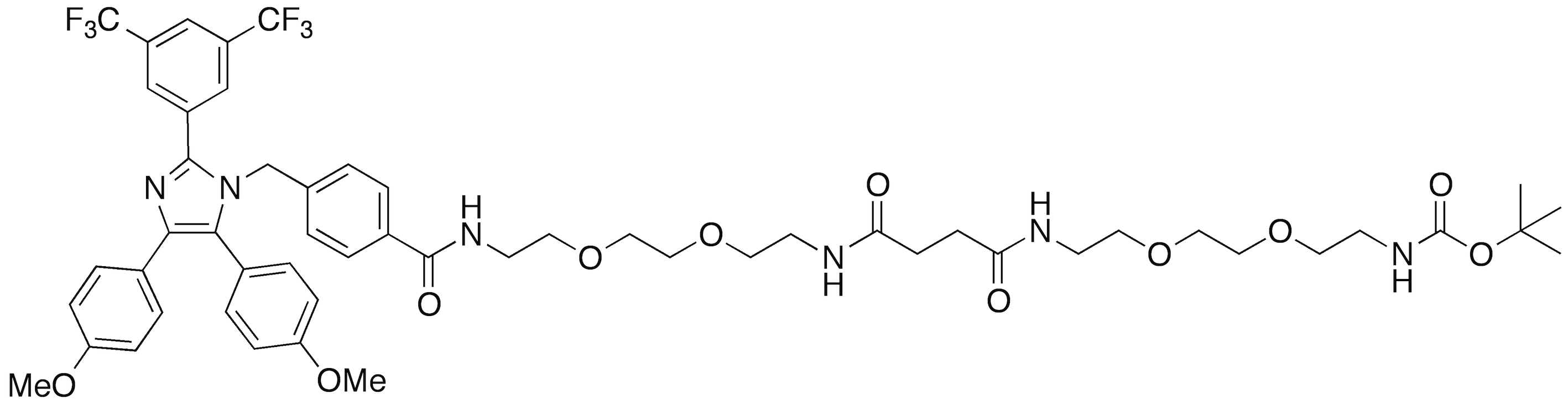


Compound **7** (0.10 g, 0.12 mmol), *N*-boc-3,6-dioxa-1,8-octanediamine (87 mg, 0.35 mmol), HBTU (51 mg, 0.14 mmol) and DIPEA (0.10 mL, 0.58 mmol) were dissolved in CH_2_Cl_2_ (4 mL) and the reaction stirred at 35 °C for 16 hours. The solvent was removed under reduced pressure and the crude product purified by semi-preparative HPLC to give the title compound (**5**) as a colourless oil (37 mg, 29%); δ_H_ (500 MHz, CD_3_OD) 8.18 (s, 2H), 8.02 (s, 1H), 7.72 (d, *J* = 8.5 Hz, 2H), 7.42 (d, *J* = 9.0 Hz, 2H), 7.26 (d, *J* = 8.8 Hz, 2H), 6.96 (d, *J* = 8.9 Hz, 4H), 6.82 (d, *J* = 9.0 Hz, 2H), 5.27 (s, 2H), 3.81 (s, 3H), 3.76 (s, 3H), 3.67 – 3.46 (m, 20H), 3.33 (t, *J* = 5.6 Hz, 2H), 3.21 (t, *J* = 5.7 Hz, 2H), 2.45 (s, 4H), 1.42 (s, 9H); δ_C_ (126 MHz, CD_3_OD) 174.7, 169.4, 161.9, 160.4, 145.8, 142.0, 139.9, 135.0, 134.1, 133.6, 133.2 (d, *J* = 33.7 Hz, 132.3, 130.3 (app d, *J* = 3.4 Hz), 129.5, 129.0, 127.4, 127.2, 125.6, 123.6, 123.4, 123.0, 115.7, 114.7, 80.2, 71.3, 71.3, 71.1, 70.6, 70.5, 55.8, 55.7, 49.3, 41.2, 40.9, 40.4, 32.2, 28.8^[[2]](#footnote-2)^; HRMS (ESI) C_54_H_66_F_6_N_6_O_11_ (M+2H)^2+^ requires 544.2341, found 544.2341.

***N*^1^-(2-(2-(2-Aminoethoxy)ethoxy)ethyl)-*N*^4^-(2-(2-(2-(4-((2-(3,5-bis(tri fluoromethyl)phenyl)-4,5-bis(4-methoxyphenyl)-1*H*-imidazol-1-yl) methyl)benzamido)ethoxy)ethoxy)ethyl)succinamide (6)**


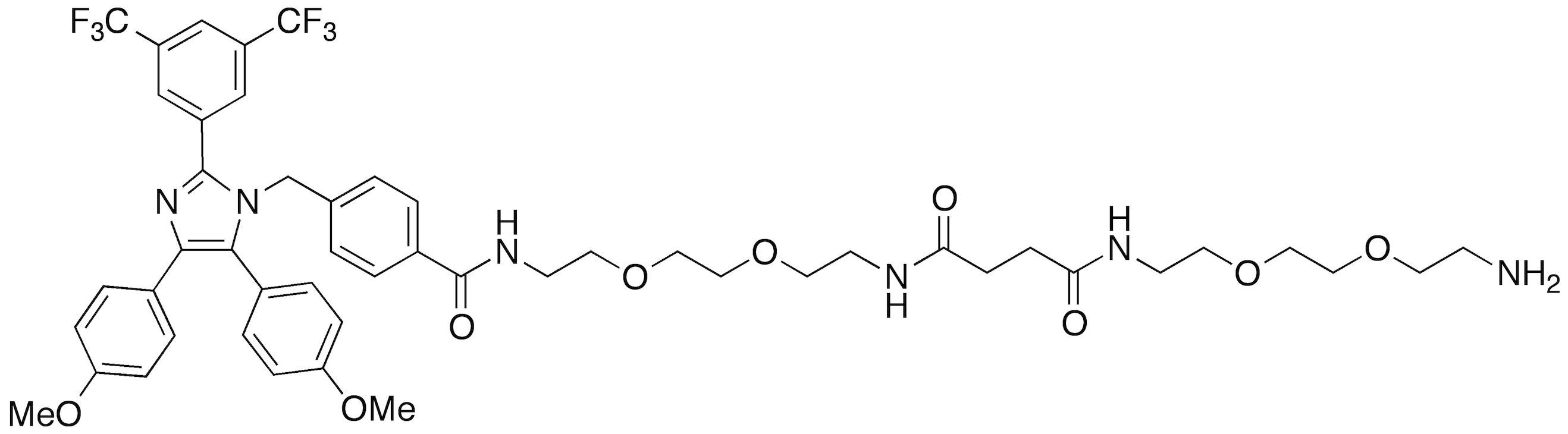


Compound **5** (10 mg, 9 µmol) was dissolved in CH_2_Cl_2_ (0.5 mL) and cooled to 0 °C, TFA (0.5 mL) was added dropwise and the reaction warmed to room temp and stirred for 16 hours. The solvent was removed under reduced pressure and the crude product purified by semi-preparative HPLC to give the title compound (**6**) as a pale yellow oil (3 mg, 33%); δ_H_ (500 MHz, CD_3_OD) 8.18 (s, 2H), 8.02 (s, 1H), 7.72 (d, *J* = 8.4 Hz, 2H), 7.42 (d, *J* = 9.0 Hz, 2H), 7.26 (d, *J* = 8.8 Hz, 2H), 6.96 (d, *J* = 8.8 Hz, 4H), 6.82 (d, *J* = 9.0 Hz, 2H), 5.27 (s, 2H), 3.81 (s, 3H), 3.76 (s, 3H), 3.67 – 3.47 (m, 20H), 3.34 (t, *J* = 5.8 Hz, 2H), 2.85 (t, *J* = 5.3 Hz, 2H), 2.45 (s, 4H); δ_C_ (126 MHz, CD_3_OD) 174.7, 169.5, 161.9, 160.4, 145.8, 142.1, 140.0, 135.0, 134.2, 133.59, 133.17 (q, *J* = 33.7 Hz), 132.3, 130.3, 129.5, 129.0, 127.5, 127.1, 123.6, 123.0, 115.6, 114.7, 72.2, 71.3, 71.3, 70.6, 70.5, 55.8, 55.7, 49.3, 41.8, 40.9, 40.4, 32.1^^[[3]](#footnote-3)^^; HRMS (ESI) C_49_H_57_F_6_N_6_O_9_ (M+H^+^) requires 987.4086, found 987.4098.

1. Marom H, Miller K, Bechor-Bar Y, Tsarfaty G, Satchi-Fainaro R, et al. (2010) Toward Development of Targeted Nonsteroidal Antiandrogen-1,4,7,10-Tetraazacyclododecane-1,4,7,10-tetraacetic Acid-Gadolinium Complex for Prostate Cancer Diagnostics. J Med Chem 53: 6316-6325. doi:10.1021/jm100289b.

2. Trester-Zedlitz M, Kamada K, Burley SK, Fenyo D, Chait BT, et al. (2003) A modular cross-linking approach for exploring protein interactions. J Am Chem Soc 125: 2416-2425. doi:10.1021/ja026917a.

3. Williams DR, Ko S-K, Park S, Lee M-R, Shin I (2008) An apoptosis-inducing small molecule that binds to heat shock protein 70. Angew Chem Int Ed 47: 7466-7469. doi:10.1002/anie.200802801.

1. C^13^ NMR spectra not obtained due to poor solubility [↑](#footnote-ref-1)
2. Several carbons not observed in spectra [↑](#footnote-ref-2)
3. Several carbons not observed in spectra [↑](#footnote-ref-3)
